# Measuring Subnational Trade Competitiveness

**DOI:** 10.1038/s41597-023-02205-z

**Published:** 2023-05-27

**Authors:** Robert A. Huber, Yannick Stiller, Andreas Dür

**Affiliations:** 1 University of Salzburg, Political Science, Salzburg, 5020 Austria; 2 University of Reading, Political Science and International Relations, Reading, RG6 6EL UK

**Keywords:** Politics, Economics

## Abstract

Much research has tried to measure the competitiveness of territorial units such as countries and subnational regions. We propose new measures of subnational trade competitiveness that reflect the economic focus of regions on their country’s comparative advantage. Our approach starts with data on the revealed comparative advantage of countries at the industry level. We then combine these measures with data on the employment structure of subnational regions to arrive at measures of subnational trade competitiveness. In total, we offer data for 6,475 regions across 63 countries and over a time period of 21 years. In this article, we introduce our measures and provide descriptive evidence, include two case studies for Bolivia and South Korea, that shows the plausibility of these measures. These data are relevant for many areas of research, including on the competitiveness of territorial units, the economic and political impact of trade on importing countries, and the economic and political consequences of globalization.

## Background & Summary

Ever since Michael Porter published a highly influential study on *The Competitive Advantage of Nations* in 1990^1^, much research has analyzed the competitiveness of territorial units. This has led to various rankings of the competitiveness of countries^2^, regions^3,4^, and even cities^5^. Over time, criticisms of the original efforts^6^ have led to various improvements in the conceptualization of territorial competitiveness^7–9^.

We contribute to this large literature by proposing new measures of what we call subnational trade competitiveness (STC). We focus on trade competitiveness at the subnational level because a measure for the trade competitiveness of a country likely hides much variation across subnational entities. In the United States, for example, the economic structure of California is substantially different from the economic structure of Louisiana, meaning that the two also likely score differently with respect to trade competitiveness. These differences across subnational regions tend to be even more pronounced in emerging economies. What is more, for individuals the trade competitiveness of the region in which they live may be more important than the one of the country as a whole, as it may better reflect their economic reality. For example, a person living in a region with a high value on trade competitiveness may experience globalization very differently from one living in the same country but in a region with low trade competitiveness. This is illustrated by the fact that support for Brexit in the United Kingdom strongly varied across regions^10^.

Our measures complement existing ones in several important ways. For one, we calculate (trade) competitiveness without recourse to potential drivers of competitiveness. Basically all existing research measures territorial competitiveness relying on input factors such as the number of people with tertiary education or the presence of certain infrastructure. Instead, we try to directly capture the ability of firms from a subnational region to sell on world markets and to compete with imports. Doing so allows for an analysis of the extent to which different factors contribute to a region’s trade competitiveness, which would be tautological when the drivers are part of the measure itself. In turn, since our measures heavily rely on trade data, we measure *trade* competitiveness rather than some overall economic competitiveness. Clearly, not all parts of an economy are exposed to international trade, even if indirectly trade matters for a larger part of the economy. Finally, exactly because we try to measure trade competitiveness rather than some broader notion of economic competitiveness, our measure needs to be independent of a region’s wealth or income per capita. By definition, all countries–and not only highly developed countries–have a comparative advantage in trade. As a result, in both more or less developed countries, regions can have economic structures more or less focused on their country’s comparative advantage. If trade competitiveness mainly captured income per capita, there would be no need for such a measure in the first place, as it would just duplicate measures such as Gross National Income.

In total, our dataset includes data for 6,475 regions in 63 countries and all continents over a 21-year period^11^. The data encompass four different measures of subnational trade competitiveness at two different aggregation levels, once at the overall and once at the sector level. In terms of sectors, we distinguish between agriculture, mining, (high-tech and low-tech) manufacturing, and services. All measures capture the average international trade competitiveness of industries in a region, which reflects the share of a region’s workforce employed in industries for which the country to which it belongs has a comparative advantage.

These data allow for research on questions as diverse as: is the anti-globalization backlash stronger in regions that see a decline in trade competitiveness? Does trade integration (for example via trade agreements) affect regions differently depending on their trade competitiveness? Which factors affect a region’s trade competitiveness? Does trade competitiveness attract foreign direct investments? Do trade shocks (for example, a sudden surge in imports, which likely affects different regions differently) have an impact on elections? Do legislators consider the economic interests of their electoral districts when casting votes? Are changes in trade competitiveness reflected in a region’s economic growth rate? Variations of our measures have already been used to address several of these questions^12–15^. As a result, the data are relevant for several strands of literature. Most directly, they can contribute to a large literature on territorial and especially regional competitiveness^4,16,17^. Moreover, a recent literature studies the consequences of the rapid increase in Chinese exports since the early 1990s on elections in the importing countries^18,19^. Others have investigated competitiveness through country-specific qualitative approaches^20^. Because our data cover many more countries than any existing measures of the China shock and is not limited to specific cases, they allow for a more comprehensive analysis of how import shocks matter for voting behaviour. Also conceptually, our measures go beyond those used in this line of research by considering exports and not only imports. The China shock studies form part of a large literature on economic geography and politics^21^ to which we add a novel measure of economic competitiveness for both developed and developing countries. Finally, our data on subnational trade competitiveness can also be used to study the economic effects of globalization. Illustratively, to which extent Greece’s entry into the Eurozone affected its economic competitiveness is a pertinent question^22^. Our measures may, for example, show that the effects of the Euro on competitiveness differ across regions.

## Methods

Our conceptual starting point is firm competitiveness, which refers to firms’ ability to sell their products on a market^23^. That is, to be competitive, firms need to produce goods and services that meet consumer demand in the markets they target and do so better than competing firms. Whether a firm is economically competitive matters strongly for its chances of survival in the market. If it is not competitive, it either goes bankrupt or requires government support to survive, for example in the form of subsidies or trade barriers that protect it from more competitive suppliers.

This logic cannot be directly transferred to territorial units^24,25^. Most fundamentally, territorial entities do not sell any goods or services on the market; only firms do. Moreover, the survival of a country or any other territorial entity does not depend on its ability to compete on markets. And whereas a firm generally needs to make a surplus, any definition that equates territorial competitiveness with the ability to achieve a balance of trade surplus is correctly criticized as mercantilist.

Building on an intense debate over these issues^9,24,26^, we thus conceive of subnational trade competitiveness as the *average international trade competitiveness of industries in a region*. By definition, each country has a comparative advantage in the production of some goods or the provision of some services. Comparative advantage results from differences in the opportunity costs of producing specific commodities across countries, which in turn are largely driven by different factor endowments. Countries that are capital-abundant generally have a comparative advantage in the production of capital-intensive goods and services; countries that are labour-abundant have a comparative advantage in the production of labour-intensive goods and services. Regions within countries vary in the extent to which they host industries for which the country has such a comparative advantage. It is this variation that we capture with our measure.

**Fig. 1:**
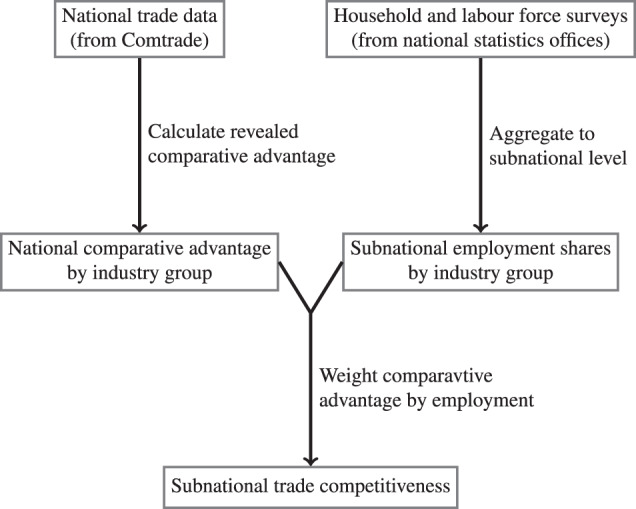
Calculation of subnational trade competitiveness.

Rather than assuming countries’ comparative advantage, we use trade data to measure their *revealed* comparative advantage (RCA) at the level of specific industries. We then use employment data from household and labour force surveys to establish to which extent a region’s economy produces goods and services for which the country has a comparative advantage. Our measure of subnational trade competitiveness hence is the combination of revealed comparative advantage at the industry-country level and region-level employment data. Figure 1 provides an illustration of our approach.

Formally, subnational trade competitiveness is calculated as follows:

1 $$ST{C}_{st}=\mathop{\sum }\limits_{g=1}^{N}\left(RC{A}_{cgt}\ast E{S}_{gst}\right),$$

where *STC*_*st*_ is the subnational trade competitiveness value for subnational region *s* at time *t*, *RCA*_*cgt*_ is the revealed comparative advantage of industry group *g* in country *c* at time *t*, and *ES*_*gst*_ is the share of region *s*’s workers across all tradeables groups employed in industry group *g* in year *t*. Larger values on the resulting measure of subnational trade competitiveness indicate a higher average international trade competitiveness of industries in a region. In other words, in a region with high values, a larger share of workers is employed in industries for which the country has a revealed comparative advantage.

Our approach rests on the key assumption that if there is a firm from a specific industry located in a specific region, its revealed comparative advantage is the same as that of another firm in the same industry in another region of the same country. This assumption is plausible because trade barriers are largely absent within countries, meaning that more competitive firms should displace less competitive firms in the same country. In other words, if there are factors that make an industry in a specific region of a country more competitive than in other regions (e.g. because of access to cheaper labour, infrastructure, or natural resources), this industry should concentrate in that region. The clustering of technology companies in Silicon Valley is a prominent example. The concentration of textile companies in one region of Austria (Vorarlberg) is a less famous, but still illustrative case.

Subnational trade competitiveness as conceptualized here captures the ability of a region’s firms to exports the goods and services they produce on world markets relative to the ability of firms from other regions in the same country. Moreover, two of the four approaches that we introduce below also capture the ability of a region’s firms to withstand international competition in domestic markets relative to firms in other regions. Overall, therefore, (changes in) subnational trade competitiveness should both reflect and affect economic and political decisions. For example, foreign firms may be more likely to invest in regions with high values on trade competitiveness. Moreover, politicians and voters in these regions may be more supportive of free trade and globalization than in other parts of the country. In turn, shocks that lead to a decline in a region’s trade competitiveness may lead to a decline in the public support that politicians in that entity enjoy. The often cited ‘China shock’^27^ is one prominent example of such shocks. There is no doubt, hence, that subnational trade competitiveness conceived of as the average international trade competitiveness of industries in a region is an important social science concept. In the following, we provide more detail on how we arrive at the RCA and employment share values that we use to calculate subnational trade competitiveness.

### Measuring a country’s (revealed) comparative advantage

Establishing the industry-level comparative advantage of a large number of countries over time is no easy feat. Illustratively, there are simply no data available on the costs of production of different goods (and the provision of different services) for many countries at different times. As a result, Balassa^28^ proposed to use trade data to measure a country’s “revealed comparative advantage”. Countries’ trade data have the advantage that they are widely available and are harmonized in terms of product classifications. They also have the disadvantage that they are affected by government policies such as trade barriers or subsidies. The “revealed” comparative advantage of a country hence may differ from the country’s actual comparative advantage. Trade liberalization, however, should have made this issue less of a concern for the period covered by our data.

Concretely, Balassa suggested standardizing the share of a specific good in total exports of a country by the given product’s share in world exports. In form of an equation:

2 $$RC{A}_{cgt}=\frac{\frac{{X}_{cgt}}{{X}_{ct}}}{\frac{{X}_{rgt}}{{X}_{rt}}},$$

where *X* refers to exports, *c* to the country, *g* to the industry group, *t* to the year, and *r* to the reference countries (e.g. the rest of the world minus country *c*, which we use in this study). Values of *RCA*_*cpt*_ above 1 indicate that a country has a comparative advantage in a given product, whereas values below 1 indicate that a country has a comparative disadvantage. This is a measure of *revealed* comparative advantage, as it takes actual trade data (which may be affected by government policies such as tariffs or subsidies) to infer comparative advantage.

Since the publication of Balassa’s paper, many authors have suggested alternative ways of calculating revealed comparative advantage (see Liu and Gao^29^ for an overview). Among the many measures that have been proposed in this debate, we choose four, all of which have the advantage that they have explicit upper and lower bounds. In fact, all four measures below have a theoretical range from −1 (greatest comparative disadvantage) to +1 (greatest comparative advantage), with a value of 0 indicating that a country has neither a comparative advantage nor a comparative disadvantage in producing a certain good or service. The other measures that have been proposed are either very similar to at least one of the measures considered here or have been shown to have serious problems.

The first RCA measure that we use is a transformation of Balassa’s original measure with the aim of making it symmetric around the neutral state of 0. Concretely, Laursen^30^ proposed the following symmetric RCA (*RCA (symmetric)*):

3 $$RCA{\left(symmetric\right)}_{cgt}=\frac{RC{A}_{cgt}-1}{RC{A}_{cgt}+1},$$

where the letters have the same meaning as in Eq. 2 above. While the fact that this measure is symmetric around 0 is a nice property of *RCA (symmetric), many of the criticisms raised against Balassa’s original RCA*^28^ *also apply to this variant. In particular, this measure generally assigns too high values* for countries and products that only account for a small share of world exports.

The second RCA measure that we rely on is an additive version of Balassa’s original measure that was proposed by Hoen and Oosterhaven^31^. This additive RCA (*RCA (additive)*) is calculated as follows:

4 $$RCA{\left(additive\right)}_{cgt}=\frac{{X}_{cgt}}{{X}_{ct}}-\frac{{X}_{rgt}}{{X}_{rt}},$$

again with the same notation as used before. According to Hoen and Oosterhaven^31^, this RCA measure has the advantage that it has a more stable distribution than the original RCA. Moreover, they criticize the original measure for having a mean above 1, although 1 should indicate the neutral point on the Balassa index. Just as *RCA (symmetric)*, *RCA (additive)* is symmetric around the neutral value of 0. A weakness of this measure, however, is that it is biased against products that only account for a small share of exports. Larger sectors receive larger values, everything else equal. This makes this measure sensitive to the level of aggregation at which it is calculated.

Because a country’s comparative advantage should be visible in both exports and imports, we also consider two RCA measures that take imports into account. Concretely, our third measure is a slight adaptation of an approach originally suggested by Vollrath^32^. Vollrath proposed subtracting a measure equivalent to the original RCA but calculated for imports (so simply substituting imports for exports in Eq. 2) from the original RCA. We adapt this approach by first applying the RCA transformation suggested by Laursen^30^ and shown in Eq. 3. In form of an equation, the resulting *RCA (net)* is calculated as:

5 $$RCA\;{(net)}_{cgt}=\left(\frac{RX{A}_{cgt}-1}{RX{A}_{cgt}+1}-\frac{RM{A}_{cgt}-1}{RM{A}_{cgt}+1}\right)/2,$$

where *RXA* refers to the RCA calculated in Eq. 2 and *RMA* to the equivalent measure calculated for imports. We divide this measure by 2 to give it the same theoretical range as the other three measures. While taking into account imports can be seen as a strength of this measure, it is at the same time also a weakness. A country may appear to have a comparative advantage for a product only because it imposes high trade barriers that strongly limit imports.

Finally, UNIDO^33^ proposed to use trade balance (which is the difference between a country’s exports and its imports) as a share of total trade as a measure of a country’s RCA. This *RCA (trade balance)* is calculated as follows:

6 $$RCA{\left(tradebalance\right)}_{cgt}=\frac{{X}_{cgt}-{M}_{cgt}}{{X}_{cgt}+{M}_{cpt}},$$

where X are exports and M imports, with the subscripts denoting the same as above. Values larger than 0 and up to 1 on this measure indicate net exports; negative values (with the lowest possible value being −1) net imports. This intuitive interpretation is a key advantage of this measure. Moreover, Leamer^34^ provides a theoretical rationale for its use as a measure of comparative advantage, as the *RCA (trade balance)* directly reflects the relative factor endowment of a country. Just as *RCA (net)*, however, this measure may be strongly affected by trade barriers.

We calculate the four RCA measures relying on data from the United Nation’s Comtrade database for trade in goods^35^ and the OECD-WTO’s BaTIS database for trade in services^36^. The goods trade data are disaggregated at the six-digit level of the Harmonised System (HS) in its 2012 version, which contains approximately 5,000 commodity groups. We have to aggregate these commodity groups to industry groups in order to match the trade with the employment data. This data is generally available at the three-digit level of the International Standard Industrial Classification of All Economic Activities (ISIC) maintained by the United Nations^37^. This level of detail is called ‘industry groups’ in ISIC jargon. Depending on the revision of ISIC (there have been three different revisions in the past decades), ISIC distinguishes between 159 and 238 distinct industry groups (e.g. beverages manufacturing). We use correspondence tables between ISIC and HS (via CPC codes) provided from the United Nations Statistics Division (https://unstats.un.org/unsd/classifications/Econ) to aggregate the goods trade data to the industry groups before calculating the four measures of revealed comparative advantage. For example, HS code 100111 (“Wheat seed for sowing”) is matched to ISIC category 0111 “Growing of cereals”.

The services trade data contains ten categories. However, we entirely focus on tradeable services, that is financial services, insurance services, and information services, thus reducing this number to three categories. Clearly, the services trade data hence are of lower quality than the goods trade data, but currently no better data are available. By offering a measure that combines the goods and services sectors and separate measures at the sectoral level, we allow researchers using our data to decide for themselves on how to deal with this limitation concerning services trade data.

All of the four RCA measures aim at capturing the same phenomenon, namely revealed comparative advantage. In fact, the correlation between them for the trade data we use is relatively high, with the partial exception of the RCA (additive). Their high correlation and the distinct strengths and weaknesses of the measures mean that we cannot say that one consistently outperforms the others. Rather, which measure works best likely depends on the question they are used to address.

### From RCA to subnational trade competitiveness

Next to the RCA on a country (*c*), industry group (*g*), and year (*t*) level (*RCA*_*cgt*_), Eq. 1 requires subnational employment data as weight (*ES*_*gst*_). We rely on household and labour surveys for this purpose. In countries where such surveys are not available, we used regular census surveys, which are conducted in five- or ten-year intervals and include the same question. While we seek to provide subnational trade competitiveness data for the 21-year period from 1999 to 2019 (with the endpoints of this period resulting from data availability), some countries do not provide labour surveys or similar in earlier parts of this time period. Thus, for some countries, we extra- or interpolated the employment share by carrying the employment data backwards and forwards. This is possible because, for the large majority of sector-specific time series (85%), the data do not show a time trend. We provide systematic tests for this in section A in the Supplementary Information. The dataset that we release contains a variable that indicates whether employment data for a specific value were imputed, so that researchers can decide themselves whether they want to use these data points. Whereas we extra- or intrapolated some of the employment shares, we always used the respective trade data for a year. In other words, even if we imputed the distribution of employees in an industry in a year, we used the correct trade data for this year.

**Fig. 2:**
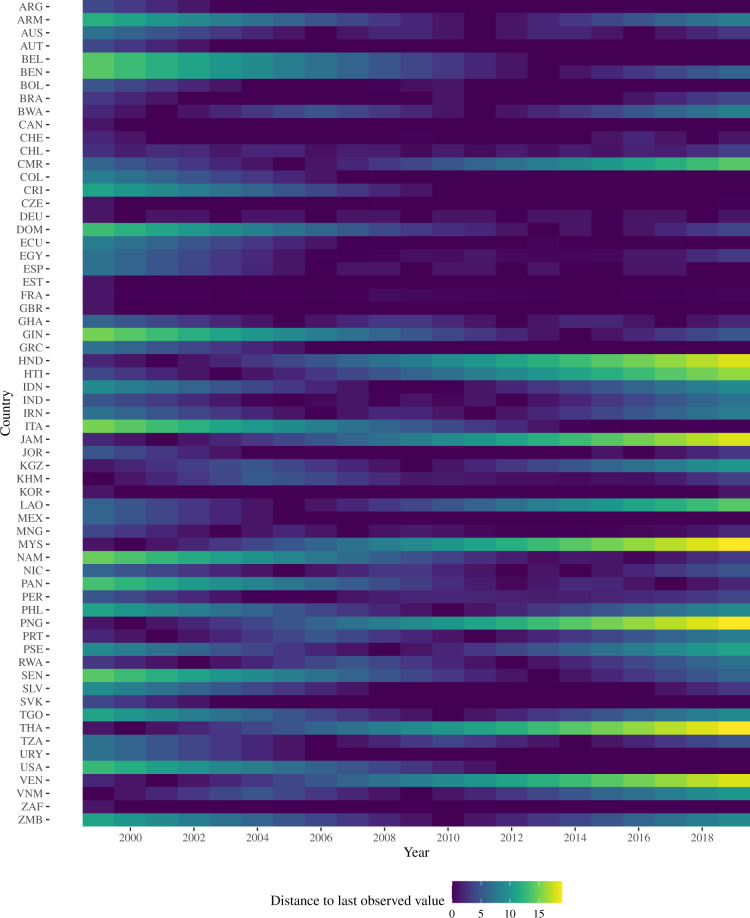
Data availability. Figure B1 in the Supplementary Information provides a map which gives a better overview of geographical coverage.

Figure 2 shows for which country-years we needed to extra- or interpolate data. Our dataset contains labour surveys for dark purple fields. The brighter the field, the larger the distance to the last labour survey. Belgium (BEL) illustrates this neatly. The Belgian Statistical Office only provides labour surveys of sufficient quality since 2013. Thus, the competitiveness estimate for 2010 uses the 2010 trade data and merges it with the first observation carried backwards, that is the 2013 employment data. For countries like Germany (DEU), we obtained data every three years. The 2016 employment data is thus a linear approximation from 2015 to 2018.

As mentioned above, this employment information is generally coded according to the ISIC scheme. In those countries that use their own adaptations of ISIC or have developed unique coding schemes, we employ official correspondence tables to transfer this data into an applicable ISIC revision. Additionally, the level of detail varies between surveys: some countries report very specific industries (coded with four or more digits) whilst others only report very broad industry sectors (coded with only two digits). To ensure both comparability and validity, we removed all surveys that use only two digits and reduce all other data to three digits. We also set all subnational trade competitiveness scores to NA for regions with fewer than 50 respondents across all tradable sectors because estimates are increasingly unreliable and unstable in smaller samples. In such cases, individual respondents are too influential and changes in competitiveness may simply reflect a marginally different composition of the sample. Figure D5 in the Supplementary Information demonstrates that this is rarely the case. With this employment data, we generated industry-group specific employment shares at the subnational level.

To determine the regional entities of interest, we generally followed the ISO 3166-2 standard. In most countries, this standard reflects the first-level administrative divisions of the country. These might be states (e.g. in Brazil, Germany, India, Mexico, and the USA), provinces (e.g. in Argentina, Belgium, Ecuador, and South Africa), or regions (e.g. in Ghana, Namibia, Peru, and Slovakia). However, in some countries, the ISO 3166-2 standard is too detailed for our purposes, which would reduce the number of survey respondents per regions to an unreliably low number. For example, this applies to the counties of Estonia and the municipalities of the United Kingdom. In these cases, we aggregated to larger statistical regions based on the European Nomenclature of Territorial Units for Statistics (NUTS) or similar. In 26 countries, the data enabled us to calculate the measure at an additional, more fine-grained level. In Italy, for example, we make available data for the more than 100 Italian provinces in addition to the 20 regions. In India, our dataset not only includes the 35 states and union territories but also the more than 600 districts. In principle, a variety of aggregation levels are possible. In total, we calculated our measure for 6,475 different regions. Table B1 in the Supplementary Information contains more information on the subnational levels provided for each country.

To calculate subnational trade competitiveness, following Eq. 1 for each industry group we then multiplied the employment share within a regional entity (which in turn is the number of workers in an industry group in a region divided by all workers in any tradeable sector within a region) by its RCA and summed the resulting products. We aggregate these measures to both the region as a whole and the region-sector level (agriculture, manufacturing (also separately for high-tech and low-tech manufacturing), mining, and services). We use the classification of the UN Statistics Division to differentiate between low-technology and high-technology manufacturing (https://unstats.un.org/unsd/classifications/Econ/Registry/Detail/3463). Given the four different ways of calculating RCA (symmetric, additive, net, and trade balance), we end up with 28 different measures for each region and year. Illustratively, we arrive at values for subnational trade competitiveness based on the symmetric RCA for a region as a whole (*STC* (*symmetric*)) or based on the net RCA for the high-tech manufacturing sector (*STC* (*net*)_*manu_high*_).

To make their interpretation more intuitive, but without this changing them per se, we subtract the national mean of the respective overall subnational trade competitiveness measure from all measures. Positive values then indicate that the average industry competitiveness of a region is higher than the one of the average region in a country. Theoretically, all of these measures range from −2 to +2, but in practice the ranges are more restricted. For all measures, Table F1 in the Supplementary Information provides summary statistics including these ranges.

The way that we calculate these measures means that absolute values may not be directly comparable across countries. For example, a value of 0.2 for Greater London does not necessarily mean that this region is twice as competitive as the region of Salto in Uruguay that has a score of 0.1. What we know is that both of these regions are more competitive than the (population-weighted) average region in these countries. The fact that our measures indicate the international trade competitiveness of a region *relative to* other regions in a country also means that not all regions in a country can gain competitiveness at the same time. If some become more competitive, others need to become less competitive.

In the end, we arrive at data for 6,475 regions in 63 countries over a 21-year period (1999–2019). Our sample of countries represents all areas of the world. For example, we capture subnational trade competitiveness for large parts of the Americas and Europe, but also for several African, Asian, and Arab countries.

Of course, there are alternatives to using employment shares as weight in Eq. 1. Compared to alternative weights that might be used to aggregate the industry-country RCA data to the subnational level, such as regional gross value added (rGVA) by industry, employment data has two crucial advantages. First, the necessary data can be obtained from household and labour force surveys, which are available for a large number of countries in a standardized way and at a highly disaggregated level. Gross value added-data by industry and subnational entity is rare. Second, weighting based on employment has conceptual benefits for many applications of this data in the social sciences that might focus on voting behaviour, political attitudes, or any broader social developments that account for the material situation of people.

Nonetheless, we investigate the extent to which our approach can be replicated by using rGVA data. Given that appropriate data on rGVA are not as readily available as employment data, we implemented this cross-check for one country from the Global North (United Kingdom) and one from the Global South (Ecuador). When calculated with this alternative data, the measures highly positively correlate with the measures based on employment data. For the United Kingdom, the correlations are between 0.92 and 0.96; and for Ecuador between 0.63 and 0.71. The lower values for Ecuador are explained by the oil industry (large value added, few employees) and agriculture (low value added, many employees). We present more detailed results in section C of the Supplementary Information. Overall, this cross-check adds confidence to our data. Section D in the Supplementary Information provides additional assessment of the data quality.

## Data Records

The dataset is stored in the Harvard Dataverse^11^: 10.7910/DVN/BWRGUR. The collection includes the dataset in two data formats, namely R’s internal data format “.rds” and comma-separated tables (“.csv”). A codebook contains central information on the variables and the data generation process. Data is also downloadable through the accompanying Shiny app: https://stcapp.shinyapps.io/STCApp/.

The data repository also includes three R-Scripts (also see section ‘Code availability’). We include three additional data sources labelled ‘mock_ISIC_schemes.xlsx’, ‘mock_industry_groups.xlsx’, and ‘mock_rca.rds’. These are necessary to perform the mock illustration outlined in the script ‘mock_calculation.R’.

The repository additionally includes a sample script that illustrates our estimation process and allows for replicating it^38^.

## Technical Validation

In this section, we start by illustrating the measurement procedure for one case, beverages in Austria. We proceed by providing descriptive evidence for our measures. Specifically, we show that the various measures of subnational trade competitiveness (for the region overall) are highly positively correlated with each other and we discuss to which extent subnational trade competitiveness varies within countries. Third, we provide several tests of the data’s face validity. Finally, we present brief case studies of South Korea and Bolivia that help better understand the data. Further analysis of the data for all measures, regions, years, and sectors is possible via the following Shiny app: https://stcapp.shinyapps.io/STCApp/.

### Illustration of measurement procedure

In the following, we provide an illustration of our measurement procedure relying on the manufacture of beverages (ISIC division 11, which is equivalent to group 110) in Austria in 2015. The first step in our measurement procedure is to establish Austria’s revealed comparative advantage in this industry group. The expectation is for Austria to be highly competitive, as it is home to the energy drink producer Red Bull. Indeed, the RCA (as outlined in Eq. 3) is 2.11, indicating that Austria exports more than twice the value of manufactured beverages than expected according to Balassa^28^. Using this information and the underlying trade data, this industry group obtains the following scores on our four measures following Eqs. 3 to 6: The symmetric RCA is 0.357, the additive RCA is 0.00609, the net RCA is 0.232 and the trade balance RCA is 0.539. In other words, all four measures indicate that Austria has a comparative advantage in the production of beverages. The additive RCA is fairly small, which is a function of the fact that manufactured beverages only account for 1.18 percent of Austrian exports in 2015. Section E in the Supplementary Information provides the detailed calculations.

The labour surveys show substantial variation in the prevalence of employment in ISIC group 110 across Austria’s regions. While only 0.02 percent of respondents in the household survey work in this group in Styria and Tyrol (after applying the survey weights), more than ten times as many work in this group in Salzburg (with 0.26 percent of respondents). That Salzburg has the highest employment share among all Austrian regions in this industry should not surprise as Red Bull has its headquarters there. Vorarlberg hosts the second largest employment share, as Red Bull’s energy drink is produced there. Again, Section E in the Supplementary Information provides more detailed information.

In sum, the trade data suggests that Austria as a country is highly competitive in the manufacture of beverages. The employment data indicates substantial variation by federal state that aligns with anecdotal evidence of where the beverage industry is located in Austria. Multiplying Austria’s RCA for this industry group with Salzburg’s employment share in this industry group hence give this region a high value. To arrive at the region’s overall trade competitiveness, we then add the scores across each industry group. In this step, hence, Salzburg’s trade competitiveness in beverages positively influences its overall competitiveness score.

### Descriptive evidence

We first present correlations between the measures of subnational trade competitiveness (as calculated for the region as a whole). The results suggest that all measures, with the partial exception of *STC*(*additive*), are highly correlated. In fact, the correlations between *STC* (*symmetric*), *STC* (*net*), and *STC* (*trade balance*) are all above 0.71 (also see Figure F1 in the Supplementary Information). The reason why *STC* (*additive*) differs a bit more from the other measures may be that it systematically plays down the role of small sectors, whereas the other measures potentially de-emphasize larger sectors. Nonetheless, the three correlations with *STC* (*additive*) are all above 0.46, indicating that all measures tap into the same latent concept (that is, subnational trade competitiveness).

**Fig. 3:**
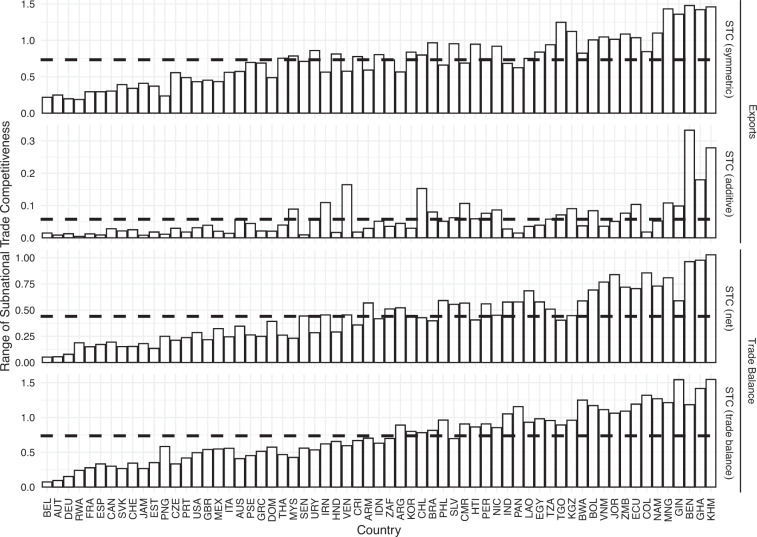
Within-country range of subnational trade competitiveness. Note: Bars are sorted by the sum of ranges across all measures.

Next we analyse to which extent subnational trade competitiveness varies *within* countries. Figure 3 shows the ranges across regions within each country. In other words, each bar shows the difference in subnational trade competitiveness between the region with the lowest and the region with the highest value on subnational trade competitiveness in a country. The colours indicate the different measures. The horizontal, dashed lines indicate the average range across all countries in our sample. Since all variables are standardised between ±1 before we subtract the country mean from them, theoretically they can take values in the range ±2. Against this theoretical range of 4, the within-country range is large. For example, the mean range for the *STC* (*symmetric*) measure is around 0.8; and hence about 20 percent of the maximum possible range. We find similar variation for the *STC* (*net*) and *STC* (*tradebalance*) measures. Only the *STC* (*additive*) operates on a more restricted part of the theoretical range.

Across all measures, we find that the countries with relatively little heterogeneity tend to be highly developed, whereas the lower income countries tend to exhibit more variation across regions. Austrian and German federal districts, for example, are similar in their employment structure, thus their level of within-country variation is rather small. The less developed countries that show up on this end tend to be relatively small, which also limits the amount of heterogeneity that exists across regions. On the other end of the extreme, we observe lower-income countries that tend to have one political and economic center and several more rural regions. A country such as Argentina that has several highly industrialised (e.g. Buenos Aires) and other more rural regions (e.g. La Pampa and Tierra del Fuego) is placed somewhere in the middle of the distribution. Again, the additive measure (*STC* (*additive*)) behaves differently from the other measures, as it produces some extreme outliers (Benin and Cambodia have five times the average range). Nevertheless, even for this measure, we find the difference between more and less highly developed countries discussed before.

### Relationship between competitiveness and GNI

Since by definition all countries have a comparative advantage in the production of some goods or the provision of some services, we should see regions with relatively high and regions with relatively low values on subnational trade competitiveness in each country. As a result, our measures cannot simply reflect cross-country differences in levels of development. Nevertheless, at the subnational level, trade competitiveness and level of development may correlate. It could be that within countries, the regions with the highest level of development also receive the highest scores on subnational trade competitiveness. If so, our measures could simply be substituted with a measure of regional GDP per capita.

**Fig. 4:**
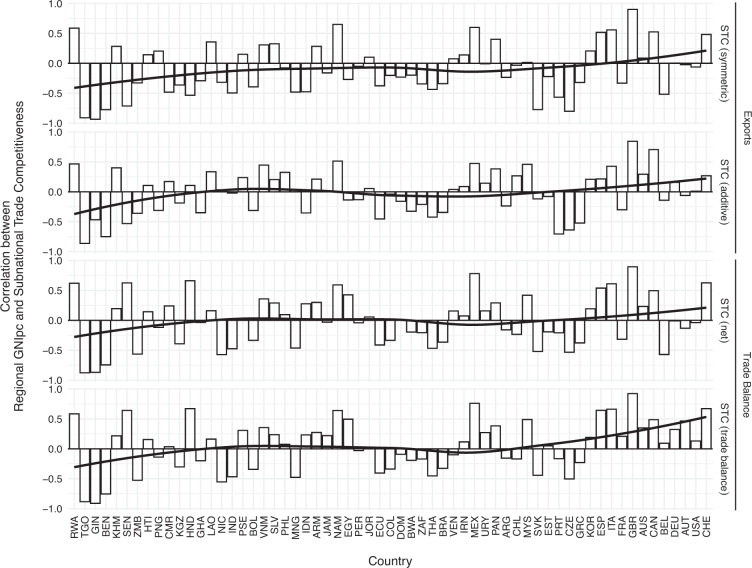
Correlation between regional GNI per capita and subnational trade competitiveness. Note: Bars are sorted by country GNI per capita.

There are good theoretical reasons to believe this is not the case, not least because low wages (which are more likely in regions with lower GDP per capita) may contribute to the comparative advantage of a country. Nevertheless, in this subsection we analyze this correlation empirically. Figure 4 shows correlations between regions’ trade competitiveness and Gross National Income per capita by country, relying on data from the Subnational Human Development Index^38^. Bars (which indicate the strength of the correlation) are sorted by the countries’ average GNI per capita over our 21-year observational period. The correlations between the two variables vary widely from quite negative to quite positive, which demonstrates that they measure different attributes of these regions. In Guinea, for example, three of four measures have a negative correlation with GNI per capita of −0.85 or below. In contrast, in Great Britain all four measures show a correlation of 0.84 or higher.

Nevertheless, we observe at least a weak pattern, as indicated by the black line that summarizes the correlations via a LOESS regression. The correlation is on average positive for the more developed countries in the sample and negative for the less developed countries. This suggests that in less developed countries, poorer subnational entities are more focused on the comparative advantage of the country, which might be explained by lower production costs (mainly wages) in these regions. In more developed countries the relationship is reversed and richer regions are more likely to produce the goods and services for which the country has a comparative advantage. This might be a result of the easier availability of both capital and high-skilled workers in wealthy districts. Overall this pattern suggests that the underlying factors that determine whether a region is oriented towards a country’s comparative advantage or not are different at different levels of development.

### Case studies: South Korea and Bolivia

In this section, we use case studies of South Korea and Bolivia to convey the intuition behind our measures. South Korea is representative of highly developed countries, whereas Bolivia reflects the situation of less developed countries. Trade data indicate that South Korea has a comparative advantage in some areas of manufacturing, especially electronic components, basic chemicals, ships, and cars. Indeed, it is home to a large electronics industry led by Samsung and LG. In addition, the Hyundai Kia Automotive Group, which is one of the world’s largest automobile producers, originates in South Korea. Hyundai Heavy Industries, Samsung Heavy Industries, and others also account for a large share of global shipbuilding (https://www.statista.com/statistics/263895/shipbuilding-nations-worldwide-by-cgt/. Our expectation is for regions in which these companies have their headquarters and/or production facilities to score highly on subnational trade competitiveness, as their economies reflect South Korea’s comparative advantage. By contrast, regions mainly characterized by other types of manufacturing or agricultural production should get low values on our measures, as overall South Korea is not particularly competitive in in these industries.

**Fig. 5:**
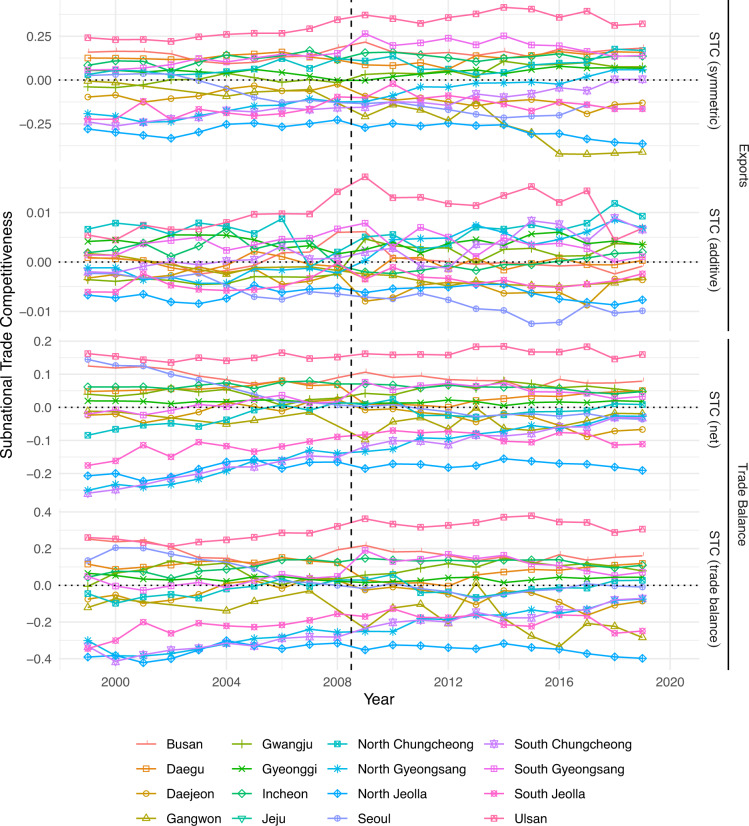
Subnational trade competitiveness of South Korean regions over time. Note: The dashed line between years 2008 and 2009 represents a change in ISIC coding scheme from ISIC rev 3 to ISIC rev 4.

Indeed, we find considerable variation in trade competitiveness across regions. In Fig. 5, the city-province of Ulsan (pink square and triangle down) consistently scores highest on trade competitiveness, across all four measures. Ulsan is home to one of the most important harbours in South Korea. It also hosts the Uslan industrial zone in which Hyundai has its headquarters and most of its production. Hence, it is clearly oriented towards South Korea’s comparative advantage. Another region that scores highly on subnational trade competitiveness is Gyeongsangnam-do (South Gyeongsang; purple square plus). This region’s economy is characterized by large shipbuilding and chemical industries. Again, these industries form part of South Korea’s comparative advantage.

The country’s capital, Seoul, used to be South Korea’s industrial centre after World War II. Decentralisation, however, led to substantial relocation of industry to other regions. The manufacturing industries that are left in Seoul are mainly in areas such as apparel and printing for which South Korea lacks a comparative advantage. Not surprisingly, Seoul does not score very high on subnational trade competitiveness, taking a place in the middle to lower end of the distribution. At the same time as Seoul’s score on subnational trade competitiveness has declined, also the importance of trade has gone down for that region, because an ever smaller share of workers has been employed in tradeables industries. The region that consistently scores lowest (North Jeolla), finally, has a relatively small manufacturing sector. In combination with a relatively large agricultural sector, this means that North Jeolla’s economy is characterized by industries for which the country lacks a comparative advantage. Figure F3 in the Supplementary Information provides more detailed evidence on these four regions that is further indication of the plausibility of our measures. For example, it demonstrates that the composition of manufacturing in the low technology sector of Seoul is out of sync with the country’s comparative advantage. In contrast, both Ulsan and South Gyeongsang’s low technology sector is highly competitive.

Contrary to South Korea, Bolivia’s comparative advantage lies in the production of crops and the mining of silver. We thus expect regions with silver mines and crop production to score highly on subnational trade competitiveness. Regions with a strong manufacturing sector, by contrast, should receive low values on subnational trade competitiveness. In fact, this is what we find (see Figure F4). The region of PotosÃ is not only Bolivia’s mining centre, as it contains the world’s largest silver deposits. It also has a large crops production. Overall, therefore, PotosÃ‘s economy is characterized by industries for which the country has a comparative advantage. It is no wonder then that this region scores highly on subnational trade competitiveness across all four measures.

In contrast, La Paz has a very different economic structure. It also has a relatively large crops production, but a substantial part of its employment is engaged in producing goods such as apparel, for which the country lacks a comparative advantage. Moreover, the mining sector in La Paz is not only smaller than the one in PotosÃ, but also does not focus on the mining of silver. In La Paz, 60 percent of employees in the mining sector are in the ISIC rev. 4 group 081 ‘Quarrying of stone, sand and clay’. The RCA values for this industry group indicate that it is not part of Boliviaâ€™s comparative advantage. In line with this anecdotal evidence, we find that La Paz’s subnational trade competitiveness is only average. The same applies to the region of Cochabamba, which is the industrial hub of Bolivia. Of the four regions shown in Figure F4, Santa Cruz scores lowest on subnational trade competitiveness, as it has much employment in animal production and services sectors for which the country’s revealed comparative advantage is strongly negative, in combination with only very limited silver mining activity.

While this between-region variation is interesting, we also observe variation over time. For this, we focus on one of the most competitive regions, Oruro (see Figure F5). Similar to PotosÃ, this region has traditionally been characterized by highly competitive silver mining. From 2004 to 2008, however, we observe a decline in its overall subnational trade competitiveness (see first dots for each year). As illustrated by the estimates for each year, while the competitiveness of individual sectors hardly changed, the region’s employment structure changed substantially. The percentage of employees working in mining decreased from more than 11.6 percent in 2004 to 2.9 percent four years later. To some extent, this can be explained by debates about the ownership of different mines, resulting in protests and eventually the closing of some mines (https://www.internationaltin.org/confrontation-at-huanuni-mine-escalates/). Thus, while mining remains fairly competitive, Oruro’s mining sector shrunk and as a result its overall trade competitiveness decreased.

## Usage Notes

The values that we get for our measures cannot be easily compared across countries, as some countries exhibit more variation across regions in terms of economic structure. This means that in heterogeneous countries the values for the most trade competitive regions are higher and the values for the least trade competitive region are lower than in more homogenous countries. Nevertheless, dichotomous versions of our measures, such as whether a region has an above-average value of trade competitiveness in a country (that is, whether the value is positive), can be meaningfully applied across countries. Moreover, the actual trade competitiveness values can be compared across regions within countries and for the same region over time. When using the absolute values in cross-national research, we strongly recommend using country fixed effects. Since the importance of international trade varies across regions, the data that we provide include a variable that indicates the share of a region’s workforce that entered the calculation of our measures.

## Supplementary information

Supplementary information

## Data Availability

The repository includes three scripts (also see section ‘Data Records’). ‘STC_calculation.R’ contains all steps from loading the raw trade data to calculating the STC. For legal reasons, we cannot share the raw data underlying the script. Table B1 in the Supplementary Information indicates the statistical offices we contacted to obtain the labour surveys. Trade data stems from the United Nation’s Comtrade database for trade in goods^35^ and the OECD-WTO’s BaTIS database for trade in services^36^. ‘Paper_replication.R’ includes the code to replicate all figures and analyses presented in this article. ‘Mock_calculation’ provides an illustration with fictious data to demonstrate each steps outlined in ‘STC_calculation.R’ with concrete data.
