# Supplementary material for: Measuring Subnational Trade Competitiveness
Source: Sci Data. 2023 May 27;10:331. doi: 10.1038/s41597-023-02205-z (PMC10224914; doi:10.1038/s41597-023-02205-z)
Supplement: Supplementary file 1 — Supplementary information [file 41597_2023_2205_MOESM1_ESM.pdf]

Supplementary Materials for ‘Measuring Subnational Trade Competitiveness’

Robert A. Huber[1,2,*], Yannick Stiller[1], and Andreas Dür[1]

[1]University of Salzburg, Political Science, Salzburg, 5020, Austria
[2]University of Reading, Political Science and International Relations, Reading, RG6 6EL, United Kingdom
[*]corresponding author(s): Robert A. Huber (robertalexander.huber@plus.ac.at)

ABSTRACT

Much research has tried to measure the competitiveness of territorial units such as countries and subnational regions. We propose new measures of subnational trade competitiveness that reflect the economic focus of regions on their country’s comparative advantage. Our approach starts with data on the revealed comparative advantage of countries at the industry level. We then combine these measures with data on the employment structure of subnational regions to arrive at measures of subnational trade competitiveness. In total, we offer data for 6,475 regions across 63 countries and over a time period of 21 years. In this article, we introduce our measures and provide descriptive evidence, include two case studies for Bolivia and South Korea, that shows the plausibility of these measures. These data are relevant for many areas of research, including on the competitiveness of territorial units, the economic and political impact of trade on importing countries, and the economic and political consequences of globalization.

Contents

A **Time Trends** **SM1**
B **Available countries and years** **SM3**
C **Regional gross value added** **SM4**
D **Data quality checks** **SM8**
E **Illustration using Austria** **SM13**
F **Additional Evidence** **SM14**
G **Used software and packages** **SM19**

A Time Trends

We systematically investigate time trends in our data. Specifically, we are interested whether there are systematic trends in the sector size within subnational entity. Strong and systematic trends would undermine our assumption to carry first observations backwards and last observations forwards. Thus, we scrutinise the subnational entity-sector changes in sector size; that is, for example, the size of the tradable service sector in the subnational region of London. To this end, we use the *funtimes* package in R[(46)]. For each district-sector combination, we investigate whether a significant monotonic trend occurs in the sector. We plot the p-values of these tests in Figure A1. By and large, we observe that only a small fraction of subnational entity-sector observations show a systematic (that is statistically significant) trend in sector size. Specifically, 14.4 per cent of all observations hold a p-value below 0.05, and there are no large differences between the sectors. High tech manufacturing has the largest share of observations with a trend (16.2 %) whereas low tech manufacturing sector has the lowest share (12.2 %). All in all, this evidence justifies carrying observations forward and backwards.

Figure A1. Distribution of time trends across sectors

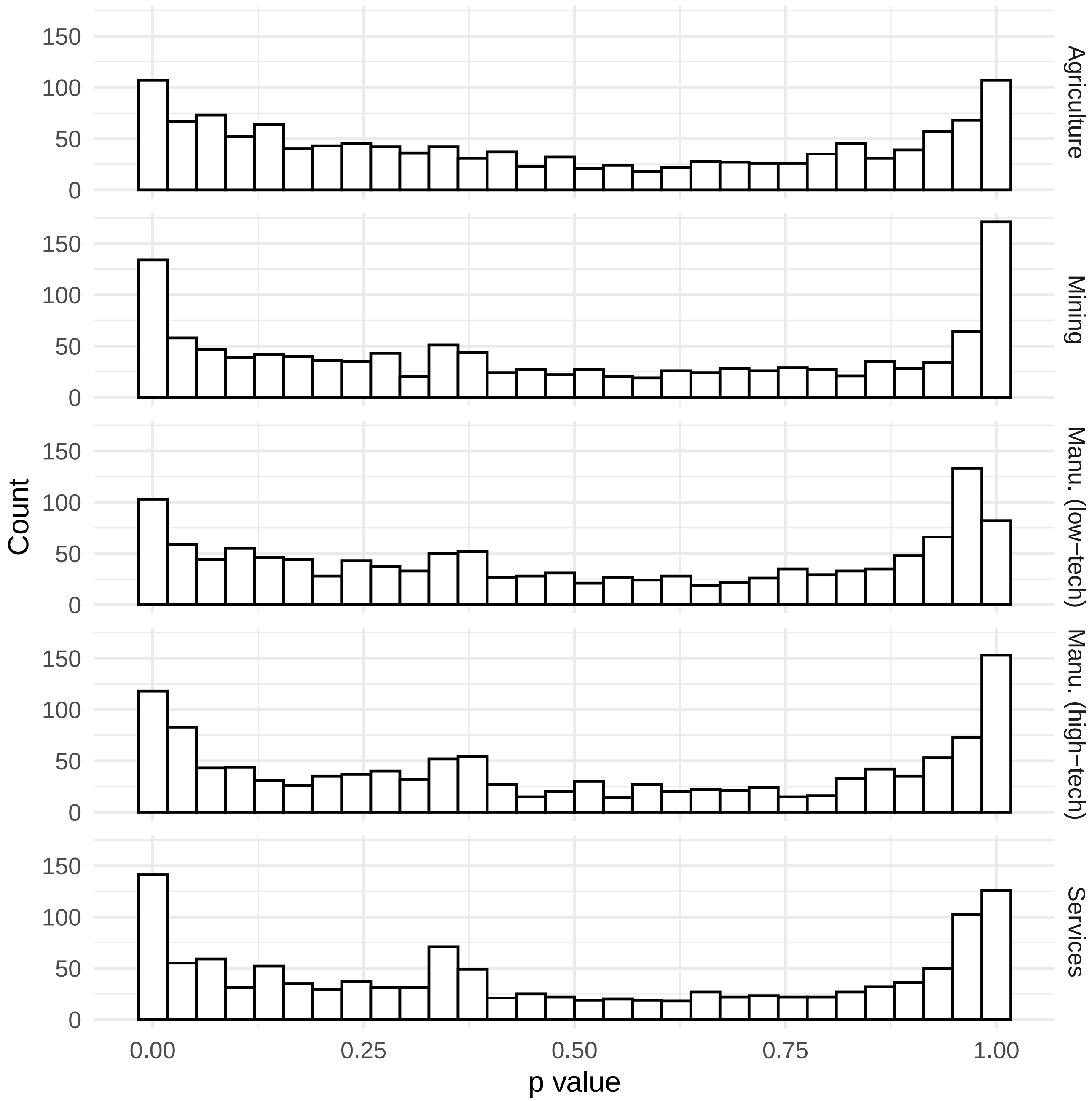

B Available countries and years

Country	Years	Regions	Coding scheme	Survey type	Source
Argentina	17	24 provinces	CAES	Household	INDEC [27]
Armenia	1	11 provinces	ISIC adapted	Census	NSS [57]
Australia	3	8 states	ANZSIC	Census	ABS [1]
Austria	17	9 states	NACE adapted	Household	StatAustria [67]
Belgium	7	11 provinces	NACE	Labor	StatBel [68]
Benin	1	12 departments	ISIC adapted	Census	INSAE [41]
Benin	1	77 communes	ISIC adapted	Census	INSAE [41]
Bolivia	15	9 departments	ISIC	Household	INE [28]
Botswana	2	10 districts	ISIC adapted	Census	StatsBots [72]
Brazil	13	27 states	CNAE	Household	IBGE [24]
Cameroon	1	10 regions	national	Census	BUCREP [8]
Cameroon	1	58 departments	national	Census	BUCREP [8]
Canada	20	10 provinces	NAICS	Labor	StatsCAN [73]
Chile	8	16 regions	ISIC	Household	MDSF [47]
Chile	8	56 provinces	ISIC	Household	MDSF [47]
Colombia	13	24 departments	ISIC adapted	Household	DANE [12]
Costa Rica	11	6 regions	ISIC	Labor	INEC [34]
Czechia	20	14 regions	NACE	Labor	CSO [10]
Czechia	20	77 districts	NACE	Labor	CSO [10]
Dominican Republic	3	10 regions	ISIC	Household	ONE [58]
Dominican Republic	2	32 provinces	ISIC	Household	ONE [58]
Ecuador	13	24 provinces	ISIC	Labor	INEC [35]
Egypt	12	29 governorates	ISIC	Labor	CAPMAS [9]
Egypt	10	325 districts	ISIC	Labor	CAPMAS [9]
El Salvador	9	14 departments	ISIC	Household	DIGESTYC [14]
Estonia	20	5 NUTS-3 regions	NACE	Labor	StatEst [69]
Germany	7	16 states	NACE	Household	DESTATIS [13]
Ghana	3	10 regions	ISIC	Household	GSS [22]
Greece	14	13 regions	NACE	Labor	ELSTAT [18]
Guinea	1	8 regions	ISIC adapted	Census	INS [40]
Guinea	1	34 prefectures	ISIC adapted	Census	INS [40]
Haiti	1	10 departments	ISIC	Census	IHSI [25]
Haiti	1	42 arrondissements	ISIC	Census	IHSI [25]
Honduras	1	18 departments	ISIC adapted	Census	INE [29]
Honduras	1	111 municipalities	ISIC adapted	Census	INE [29]
India	7	35 states	ISIC	Labor	MoSPI [49]
India	6	625 districts	ISIC	Labor	MoSPI [49]
Indonesia	14	33 provinces	KBLI	Labor	BPS [7]
Iran	2	31 provinces	ISIC adapted	Census	SCI [65]
Iran	2	394 counties	ISIC adapted	Census	SCI [65]
Italy	6	20 regions	NACE	Labor	ISTAT [43]
Italy	6	105 provinces	NACE	Labor	ISTAT [43]
Jamaica	1	14 parishes	ISIC adapted	Census	STATIN [70]
Jordan	12	12 governorates	ISIC	Labor	DOS [15]
Kyrgyzstan	2	9 regions	ISIC adapted	Census	NSC [53]
Laos	1	18 provinces	ISIC adapted	Census	LSB [45]
Malaysia	1	15 states	ISIC	Census	DOSM [16]
Malaysia	1	133 districts	ISIC	Census	DOSM [16]
Mexico	15	32 states	SCIAN	Labor	INEGI [37]
Mongolia	13	5 regions	ISIC	Labor	NSO [54]
Mongolia	13	22 provinces	ISIC	Labor	NSO [54]
Namibia	2	15 regions	ISIC	Labor	NSA [52]
Nicaragua	3	17 departments	ISIC	Household	INIDE [39]
Palestinian Territories	1	16 governorates	ISIC	Labor	PCBS [60]
Panama	2	13 provinces	ISIC adapted	Labor	INEC [36]
Papua New Guinea	1	20 provinces	ISIC adapted	Census	NSO [56]
Papua New Guinea	1	87 districts	ISIC adapted	Census	NSO [56]
Peru	16	26 regions	ISIC	Household	INEI [38]
Peru	16	195 provinces	ISIC	Household	INEI [38]
Philippines	1	17 regions	ISIC adapted	Census	PSA [62]
Philippines	1	80 provinces	ISIC adapted	Census	PSA [62]
Portugal	2	7 NUTS-2 regions	NACE	Census	INE [33]
Rwanda	2	5 provinces	ISIC	Census	NISR [51]
Senegal	1	14 regions	ISIC adapted	Census	ANSD [2]
Senegal	1	45 departments	ISIC adapted	Census	ANSD [2]
Slovakia	17	8 regions	NACE	Labor	StatSVK [74]
South Africa	20	9 provinces	SIC	Labor	Stats SA [71]
South Korea	19	17 provinces	KSIC	Labor	KLI [44]
Spain	7	19 communities	NACE	Labor	INE [30]
Switzerland	15	7 regions	NACE	Labor	BfS [6]
Switzerland	15	26 cantons	NACE	Labor	BfS [6]
Tanzania	2	25 regions	ISIC	Labor	NBS [50]
Thailand	1	5 regions	ISIC	Census	NSO [55]
Thailand	1	76 provinces	ISIC	Census	NSO [55]
Togo	1	6 regions	ISIC adapted	Census	INSEED [42]

Togo	1	37 prefectures	ISIC adapted	Census	INSEED[42]
United Kingdom	20	12 NUTS-1 regions	NACE	Labor	ONS[59]
United States	20	51 states	US Census	Household	USCB[76]
Uruguay	14	19 departments	ISIC	Household	INE[31]
Venezuela	1	24 states	ISIC	Census	INE[32]
Venezuela	1	237 municipalities	ISIC	Census	INE[32]
Vietnam	2	8 regions	ISIC adapted	Census	GSO[21]
Vietnam	2	64 provinces	ISIC adapted	Census	GSO[21]
Zambia	1	74 districts	ISIC adapted	Census	ZamStats[82]

Table B1. Summary of available countries

The surveys for Armenia, Benin, Botswana, Cameroon, Guinea, Haiti, Iran, Italy, Kyrgyzstan, Laos, Malaysia, Papua New Guinea, the Philippines, Portugal, Rwanda, Senegal, Thailand, Togo, the USA, Venezuela, Vietnam, and Zambia were provided by the Minnesota Population Center[48]. The surveys for Egypt, Jordan, and the Palestinian Territories were provided by the Economic Research Forum[17].

Figure B1. Available countries

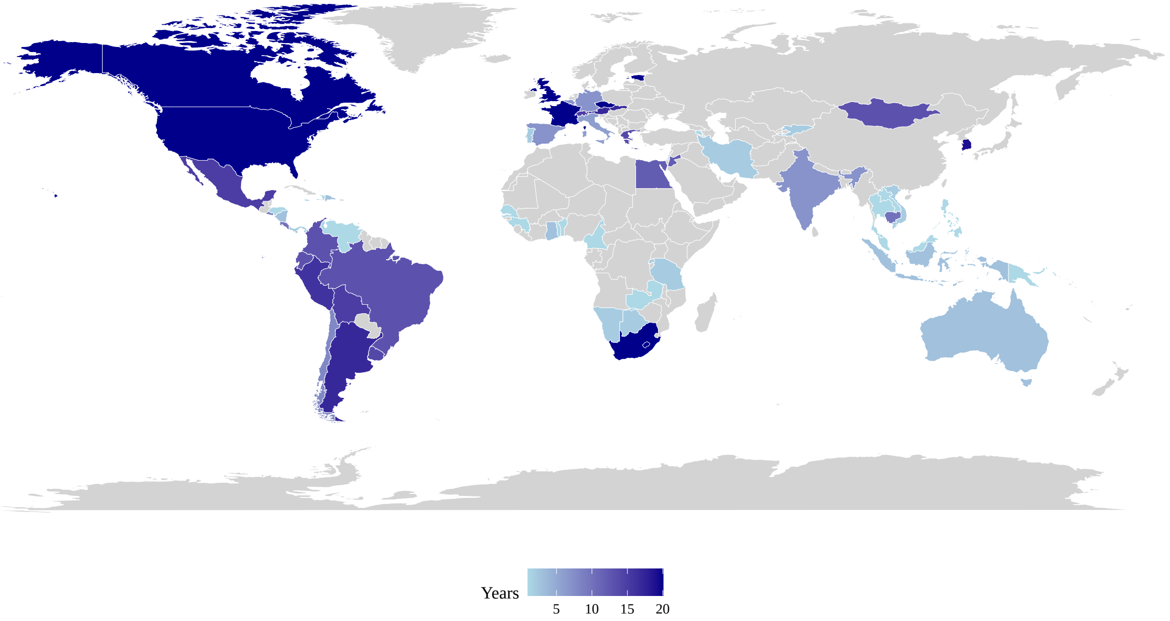

C Regional gross value added

The STC measure is a weighted mean of RCA measures. Equation 1 emphasises this. In other words, ES_{gst} is an weighted which allows to transform the country-level RCA for an industry in a given time (RCA_{cgt}) to a measure on a subnational level (namely STC_{st}).

As discussed in the main-text, employment shares through labour surveys are widely available and hold important advantages to other measures. On prominent alternative are gross-value-added measures (GVA). In order to be an alternative, these measures have to be available on a regional level and fine-grained in terms of industry groups. We collected data for two countries, the United Kingdom (as a developed economy) and Ecuador (as a developing economy) and compare the STC scores and weights.

Figures C1 and C2 plot the resulting STC scores based on labour survey weights on the x-axis and the GVA scores respectively on the y-axis. Each dot is one STC value, thus a subnational entity in a given year. The black line indicates a linear fit between both variables. The figures by and large suggest that scores strongly correlate. London is by far the most competitive district (top right of all four facets), whereas other districts are less focused on industries for which the UK has a comparative advantage. In Ecuador, the picture is generally similar. However, at least some districts are highly competitive in GVA-based measures but less so in labour survey-based measures. This reflects that these districts export oil, an industry with a small workforce (meaning that it gets a small weight according to labour surveys) but a large gross value added.

Figures C3 and C4 plot the underlying survey weights to ensure that the previous findings are not entirely driven by the aggregation. Thus, these figures plot the labour survey weights on the x-axis and the gva weights on the y-axis. Each dot is a industry group (g in equation 1) in a subnational entity in a given year. The black line again indicates a linear fit between both variables. Figure C3 shows that generally the weights follow fairly similar trajectories. Figure C4 confirms this notion, but also emphasises that some industry groups receive a small weight according to employment data but a high weight according to the GVA (see top left quadrant of Figure C4).

All in all, these findings suggest that our decision to use labour surveys does not result in substantially different results than the use of less-widely available GVA data would yield.

Figure C1. Comparison of labour surveys-based and GVA-based STC scores in the United Kingdom

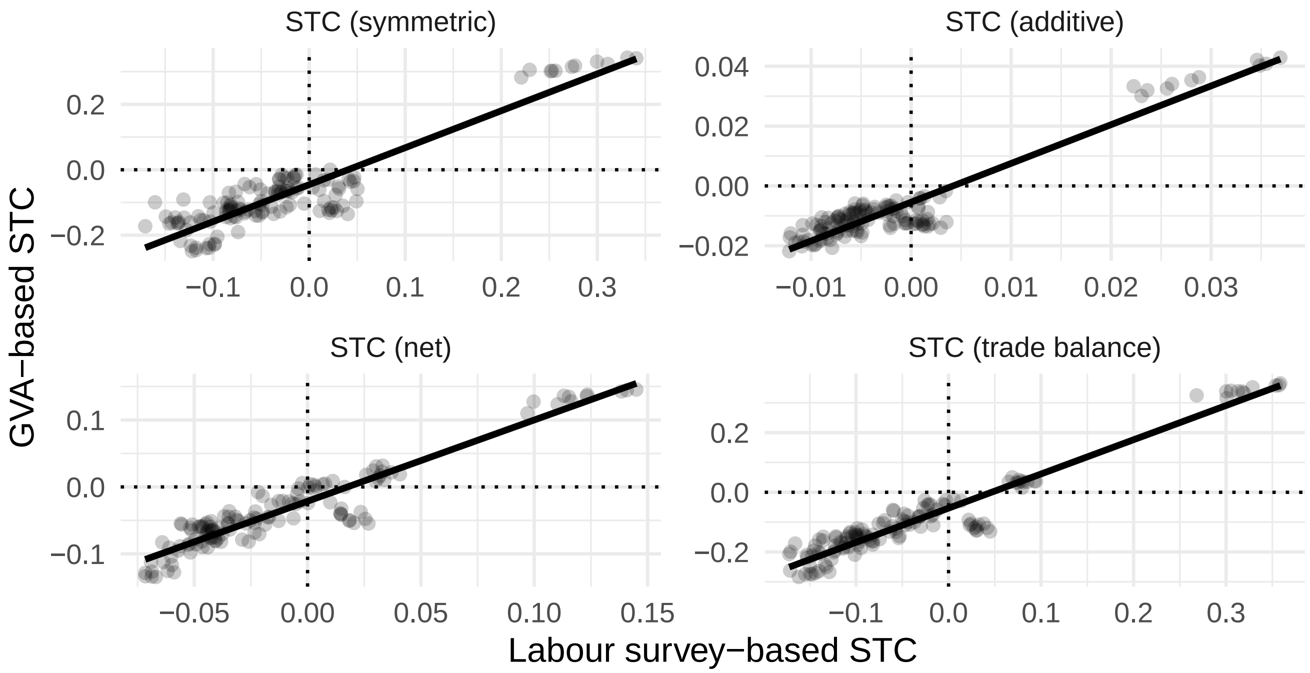

Figure C2. Comparison of labour surveys-based and GVA-based STC scores in Ecuador

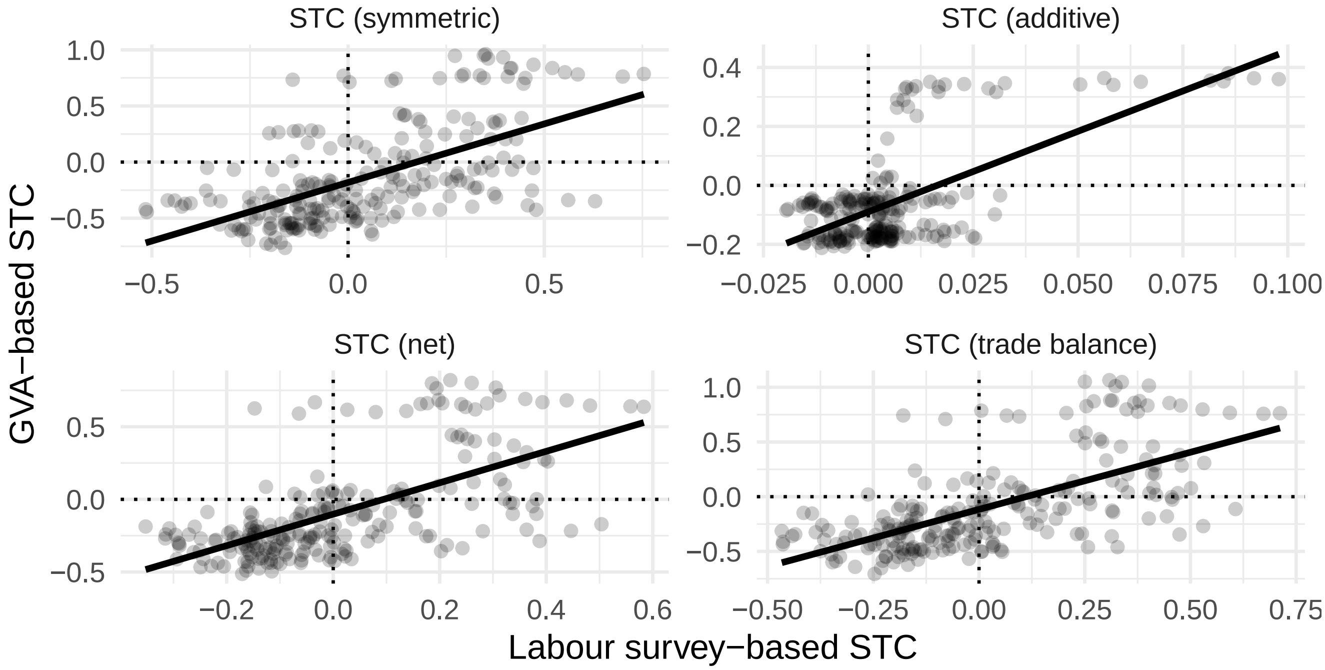

Figure C3. Comparison of labour surveys-based and GVA-based weights in the United Kingdom

Figure C4. Comparison of labour surveys-based and GVA-based weights in Ecuador

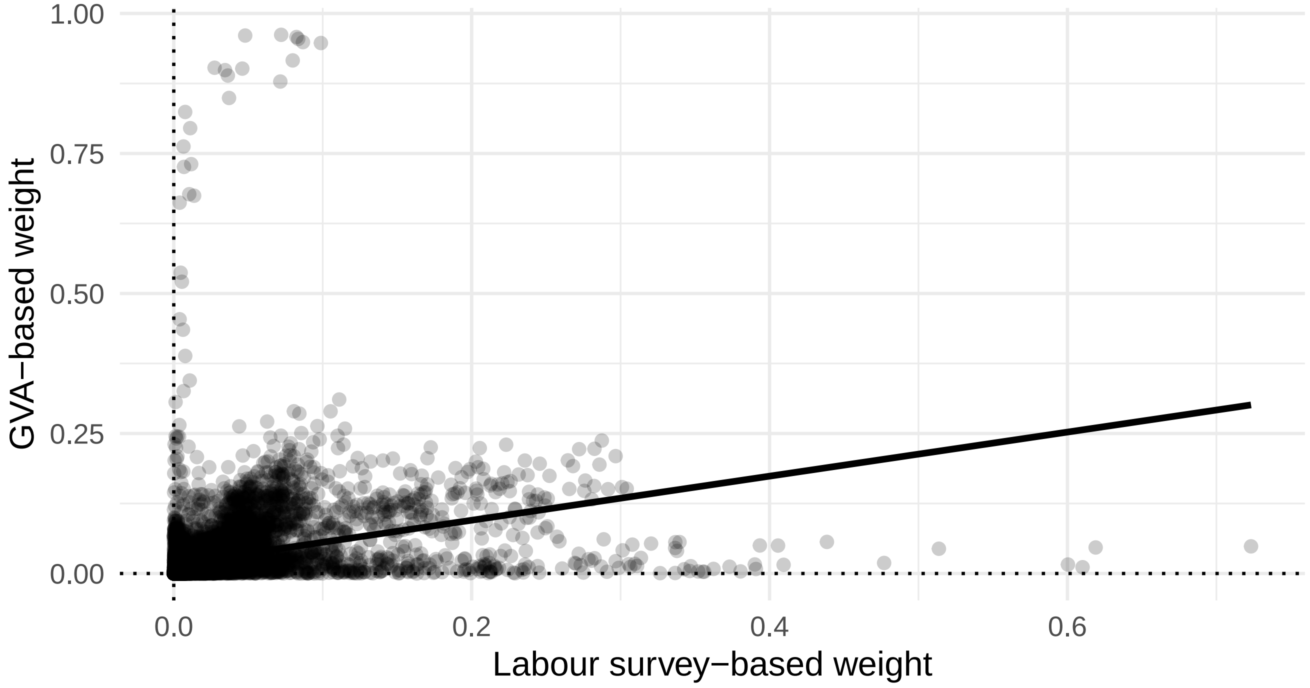

D Data quality checks

This sections provides some quality checks of the labour and household surveys that we use to assess the regional distribution of industries within a country. These checks demonstrate that overall the data quality of the labor and household surveys is very good and suited for our purposes. We perform the following five tests:

- Figure D1: Does the aggregated data yield the correct industry sector shares of employment on the national level? This ensures that no industry sector is over- or underrepresented. We compare our data to the estimates of sector employment of the International Labour Organization (ILO)[(26)].
- Figure D2: Does the aggregated data yield the correct population shares for each region within the country? This ensures that no region is over- or underrepresented. We compare our data to population data of the Subnational Human Development Index (SHDI)[(66)].
- Figure D3: Is the original data coded correctly? This ensures that we do not use incorrectly coded data or data that uses a different coding scheme than assumed. We compare our data to the coding schemes of the International Standard Industrial Classification (ISIC)[(75)].
- Figure D4: Is transferring the original coding scheme into the corresponding ISIC scheme causing too much duplication? When the original coding scheme is not as detailed as the ISIC scheme, some respondents are attributed to more than one ISIC category. This duplication should be kept to a minimum.
- Figure D5: Is the aggregated data based on enough respondents? This ensures that our data is robust.

Figure D1. Employment shares by national industry sector

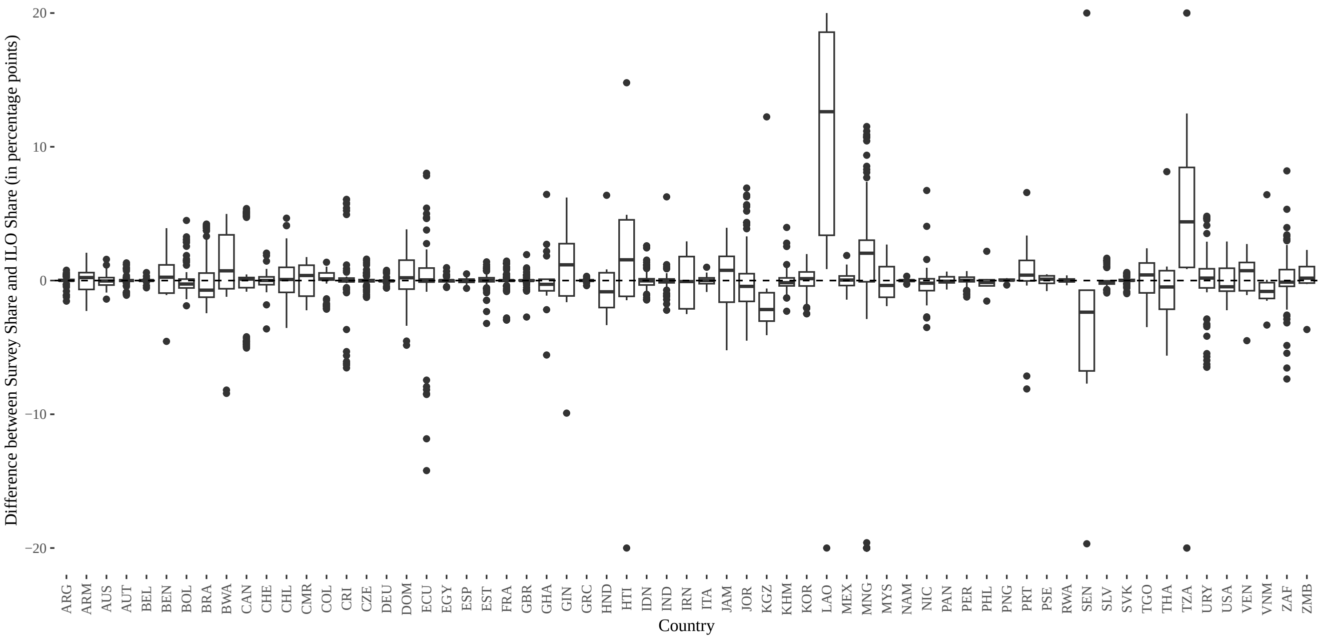

Ideally, the shares should match exactly. In some countries such as Laos and Tanzania, subsistence pastoral farmers are not included in the labour surveys, which explains the large difference to the ILO data.

Figure D2. Population shares by region

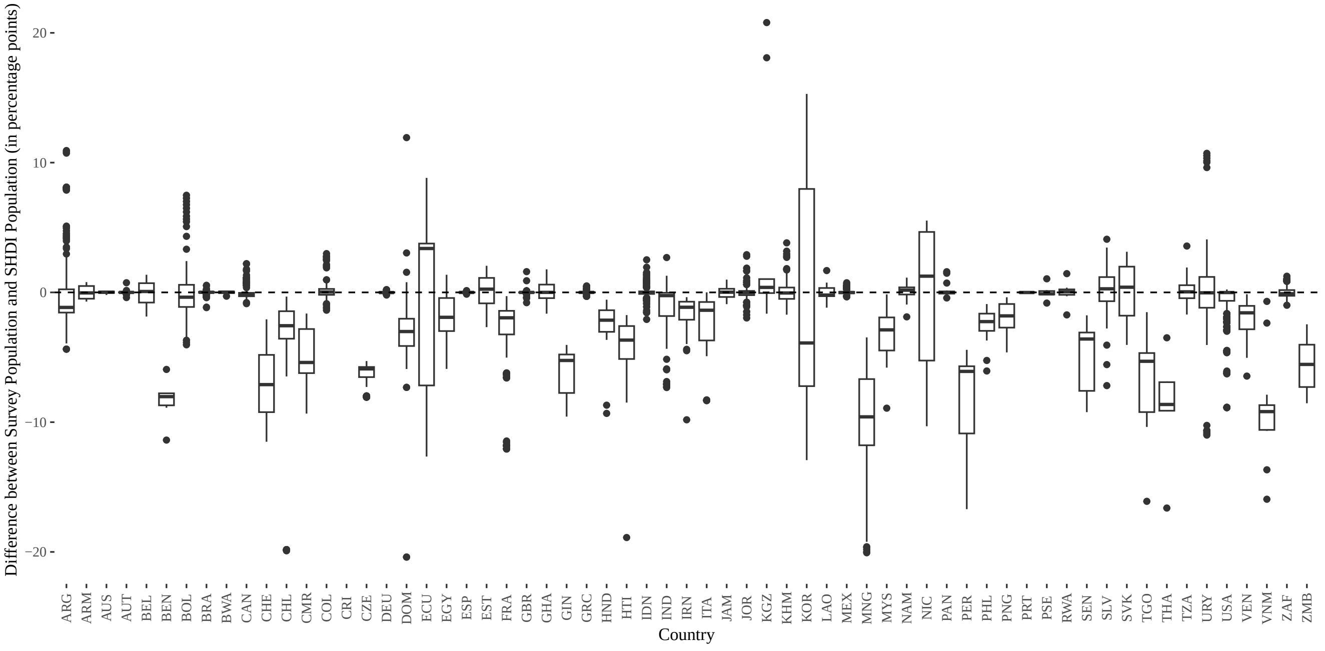

Ideally, the shares should match exactly. For most regions, the shares we have closely resemble the population shares calculated from the SHDI dataset.

Figure D3. Share of incorrect industry codes

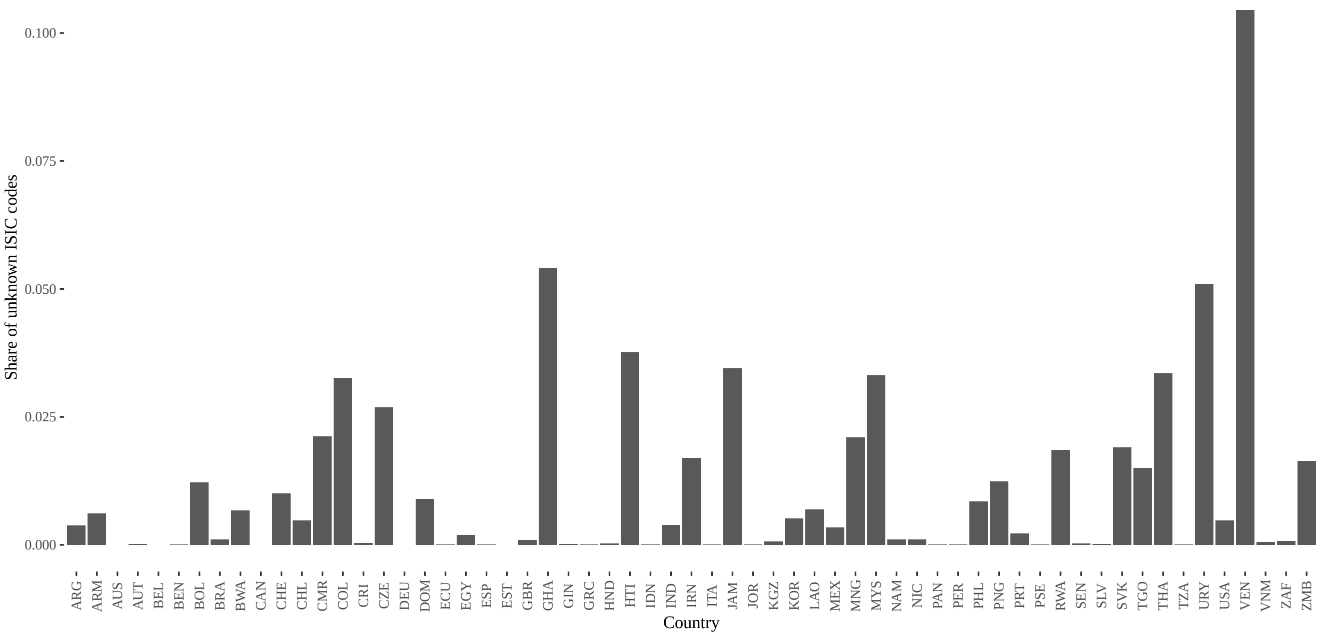

Ideally, there should be no incorrect ISIC codes. However, some countries use special categories that cannot be transferred into standard ISIC schemes. An example is Bolivia, which has a unique code for professional football players. Other countries suppress the codes of certain respondents. We treat these codes as NAs.

Figure D4. Ratio of duplicated respondents

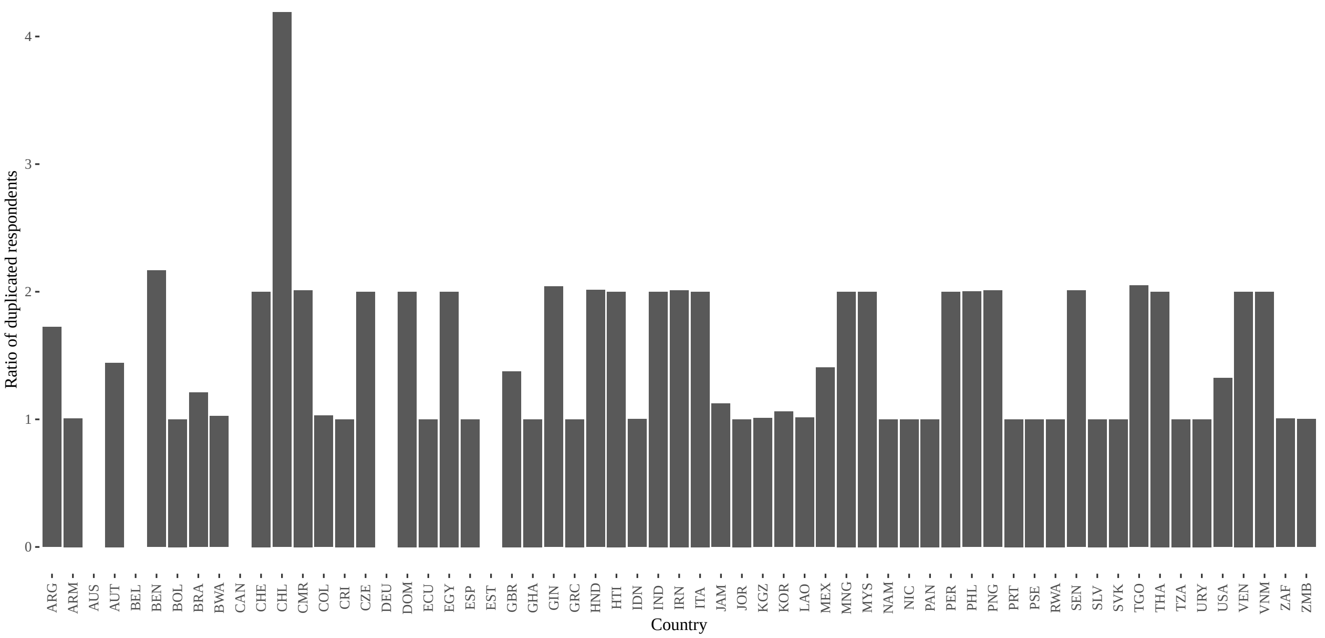

When the original coding scheme is different than the standard ISIC schemes, we use official correspondence tables. Sometimes, these original coding schemes are not as detailed as the corresponding ISIC scheme, which causes the duplication of respondents (who will be attributed to more than one ISIC category). The ratio should ideally be 1, which means that there are no duplicates. In Australia, Belgium, Canada, Germany, and Estonia, the calculation of duplicates is not possible due to technical reasons. However, the ratio of duplicates is close to 1 in these countries.

Figure D5. Share of regions with few workers

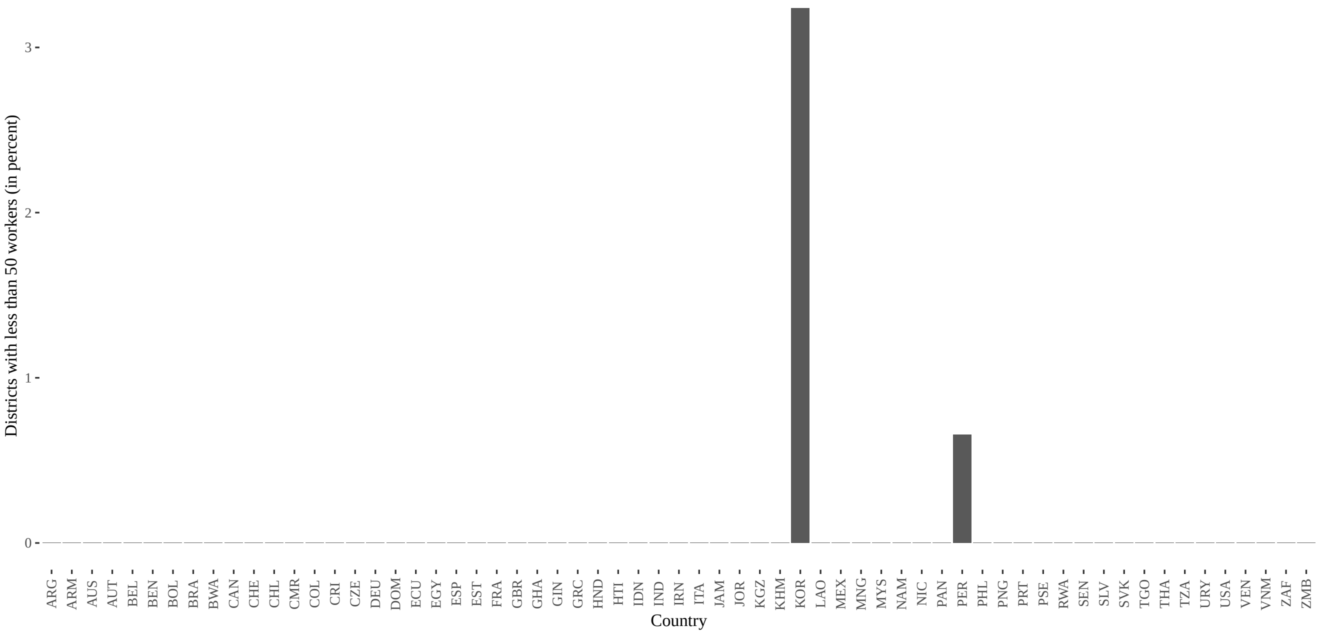

Ideally, the survey would include a large number of workers for each region. However, this is not the case in countries with many first level administrative divisions relative to their population such as Switzerland or Cambodia. In Korea, the labor survey is a panel survey with a low number of respondents. We drop regions if we have fewer than 50 workers to calculate the competitiveness measures.

E Illustration using Austria

This section provides additional information on the measurement illustration for the ISIC group 'Manufacture of Beverages' (110) in Austria in 2015. Figure E1 provides the underlying trade data and its aggregations. The first eight entries (from 'Country exports in ISIC group 110' to 'World total imports') represent the trade flows from and to Austria in million US$. For example, Austria exports products worth 2.233 billion US$. Imports in this industry amount to 668 million US$. This data is used to estimate different measures of revealed comparative advantages. The comparison of the variables 'Share of country exports' (1.18) and 'Share of world exports' (0.56) already indicates that Austria is exporting substantially more than expected based on all other countries in the world. This directly results in the 'Exports based RCA (RXA)' of 2.11, which is well above the neutral value of 1. Similarly, the 'Imports based RCA (RMA)' indicates that this industry faces relatively fewer imports than the average country. This is again reflected in the positive RCA scores across all four operationalisations.

Table E1. Trade data and measures for Austria

Variable	Value	Eq. 2	Eq. 3	Eq. 4	Eq. 5	Eq. 6
Country exports in ISIC group 110	2233.80	X		X	O	X
Country imports in ISIC group 110	668.34				O	X
Country total exports	191934.35	X		X	O	
Country total imports	183901.22				O	
World exports in ISIC group 110	128579.95	X		X	O	
World imports in ISIC group 110	103372.76				O	
World total exports	23155297.92	X		X	O	
World total imports	22929514.25				O	
Share of country exports	1.18	O		O	O	
Share of world exports	0.56	O		O	O	
Share of country imports	0.36				O	
Share of world imports	0.45				O	
Exports based RCA (RXA)	2.11		X		X	
Imports based RCA (RMA)	0.81				X	
RCA(symmetric)	0.36					
RCA(additive)	0.01					
RCA(net)	0.23					
RCA(trade balance)	0.54					

Entries directly derived from trade data (from "Country exports in ISIC group 110" to "World total imports" are in million US$. Share values are in percent. 'X' indicate that this variable is directly used in the respective equation in the main paper. 'O' indicates that this variable is indirectly used as an ancillary concept in an equations (for example expressed through the 'X' variables.

Table E2 shows the percentage of the workforce in each of Austria's nine regions employed in ISIC group 110. As described in the main text, Salzburg and Vorarlberg have a large beverage industry, whereas this industry is much weaker in the other states.

Table E2. Labour Survey Data for ISIC Group 110 (2015)

Subnational code	Subnational name	Respondent percentage	Weighted percentage
AT-1	Burgenland	0.45	0.14
AT-2	Carinthia	0.34	0.13
AT-3	Lower Austria	0.21	0.07
AT-4	Upper Austria	0.17	0.05
AT-5	Salzburg	0.64	0.26
AT-6	Styria	0.09	0.02
AT-7	Tyrol	0.07	0.02
AT-8	Vorarlberg	0.48	0.19
AT-9	Vienna	0.14	0.05

The subnational code corresponds to the ISO 3166-2 standard. Respondent percentage is calculated using the number of individuals within the labour survey in the ISIC group 110. Weighted percentage is the percentage of employment after applying survey weights.

F Additional Evidence

Figure F1. Correlation between the four measures

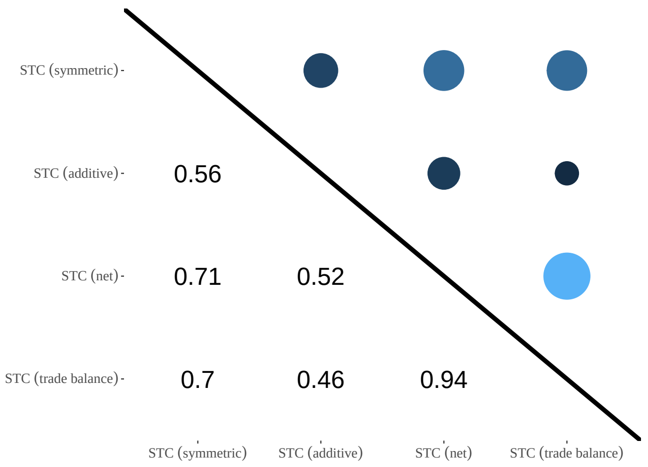

Figure F2. Correlation between the four measures before aggregation with labour surveys

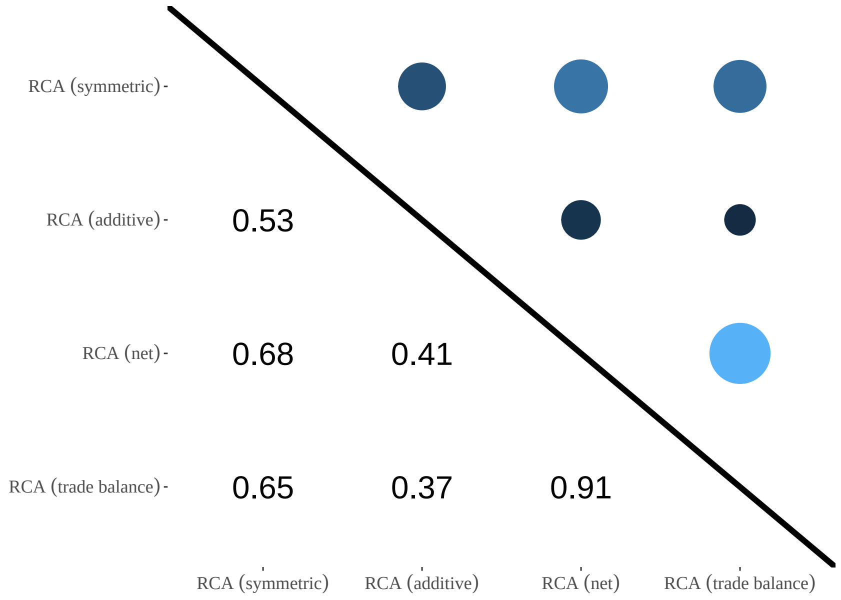

Table F1. Summary statistics of all STC measures

Statistic	N	Mean	St. Dev.	Min	Max
STC (symmetric)	130,572	0.0000	0.15	−1.30	1.07
STC (additive)	130,572	0.0000	0.02	−0.28	0.48
STC (net)	130,572	−0.002	0.10	−0.83	0.88
STC (trade balance)	130,572	−0.003	0.16	−1.39	1.11
STC (symmetric agri)	129,385	0.06	0.26	−1.13	1.29
STC (additive agri)	129,385	0.0003	0.01	−0.22	0.12
STC (net agri)	129,385	0.12	0.24	−1.00	0.89
STC (trade balance agri)	129,385	0.20	0.41	−1.52	1.20
STC (symmetric mini)	108,078	−0.15	0.69	−1.59	1.89
STC (additive mini)	108,078	0.02	0.13	−0.28	0.80
STC (net mini)	108,078	0.04	0.47	−1.27	1.47
STC (trade balance mini)	108,078	−0.02	0.69	−1.64	1.87
STC (symmetric manu)	129,209	−0.04	0.32	−1.63	1.58
STC (additive manu)	129,209	−0.004	0.03	−0.28	0.35
STC (net manu)	129,209	−0.01	0.18	−1.30	1.04
STC (trade balance manu)	129,209	−0.04	0.33	−1.71	1.22
STC (symmetric manu lt)	129,171	−0.05	0.32	−1.66	1.59
STC (additive manu lt)	129,171	−0.01	0.02	−0.28	0.35
STC (net manu lt)	129,171	−0.01	0.19	−1.36	1.04
STC (trade balance manu lt)	129,171	−0.04	0.34	−1.73	1.22
STC (symmetric manu ht)	114,919	−0.11	0.39	−1.72	1.22
STC (additive manu ht)	114,919	−0.01	0.03	−0.30	0.34
STC (net manu ht)	114,919	−0.10	0.20	−1.46	0.71
STC (trade balance manu ht)	114,919	−0.19	0.34	−1.78	1.16
STC (symmetric serv)	122,944	−0.28	0.41	−1.73	1.27
STC (additive serv)	122,944	−0.01	0.03	−0.31	0.11
STC (net serv)	122,944	−0.04	0.19	−0.88	0.82
STC (trade balance serv)	122,682	−0.04	0.37	−1.50	1.50

Figure F3. Sectoral competitiveness by region in South Korea

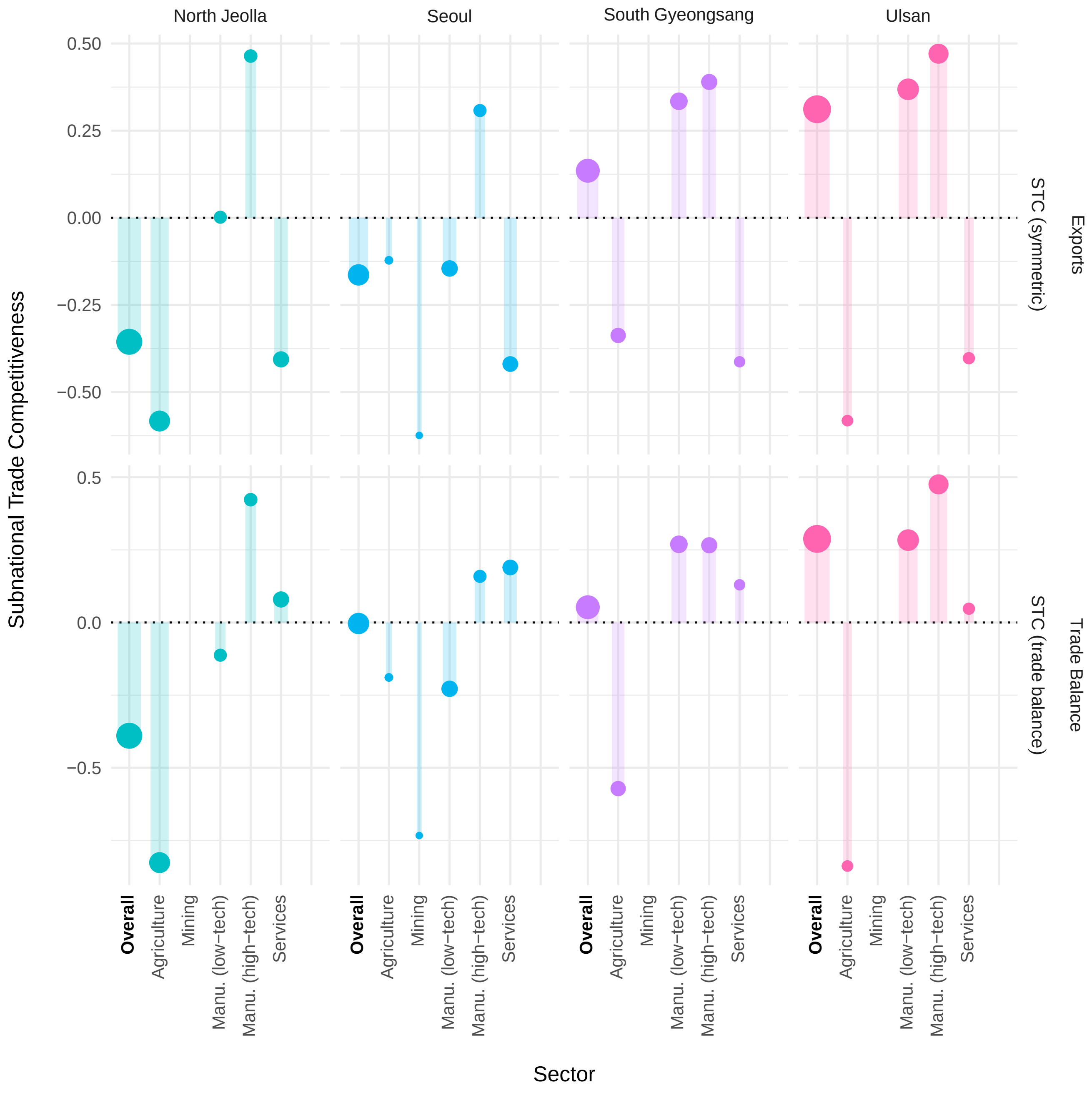

Note: Data for the year 2018.

Figure F4. Subnational trade competitiveness of Bolivian regions over time

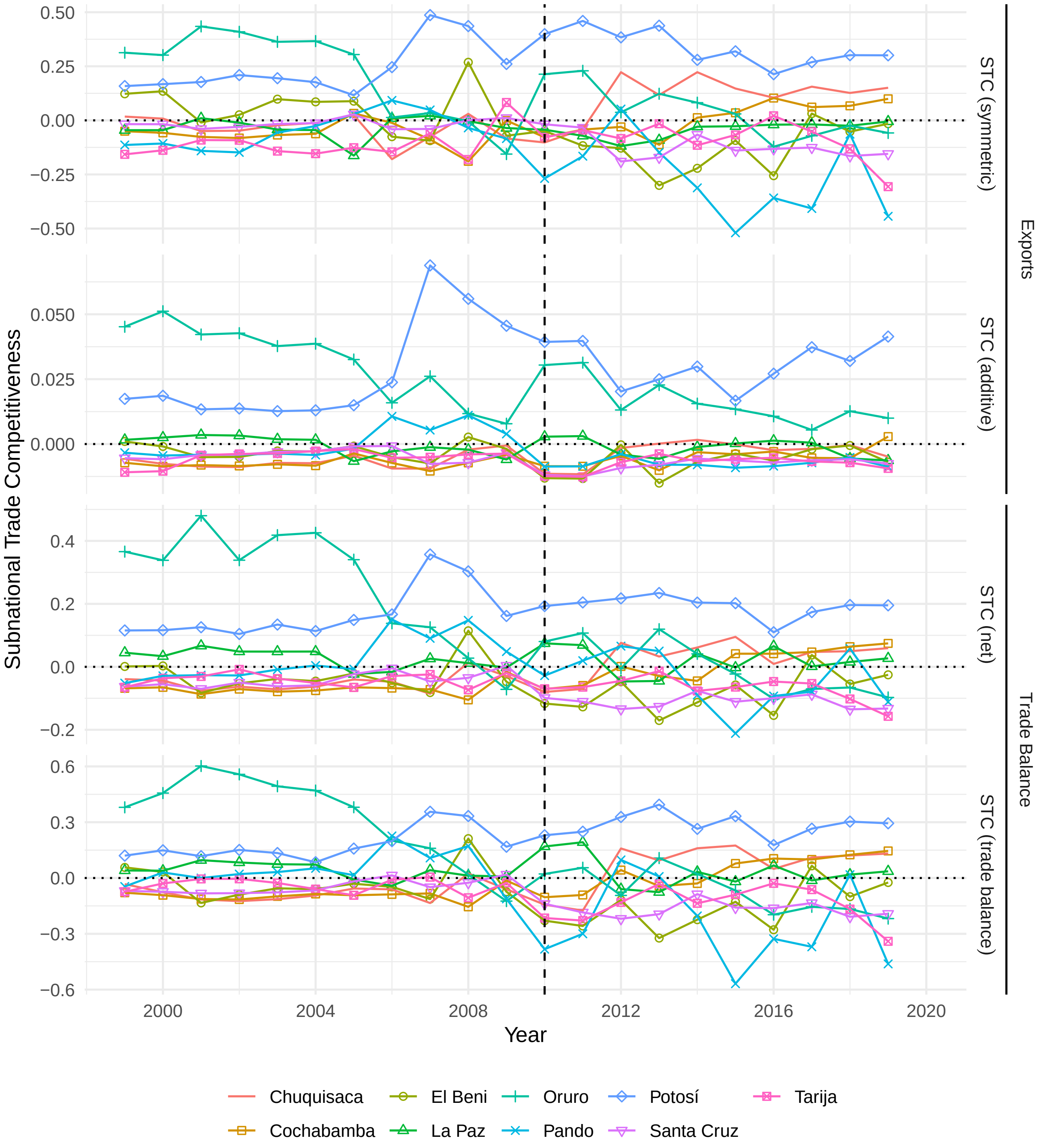

Note: The dashed line in 2009 represents a change in ISIC coding scheme from ISIC rev 3.1 to ISIC rev 4.

Figure F5. Sectoral competitiveness by region in Bolivia

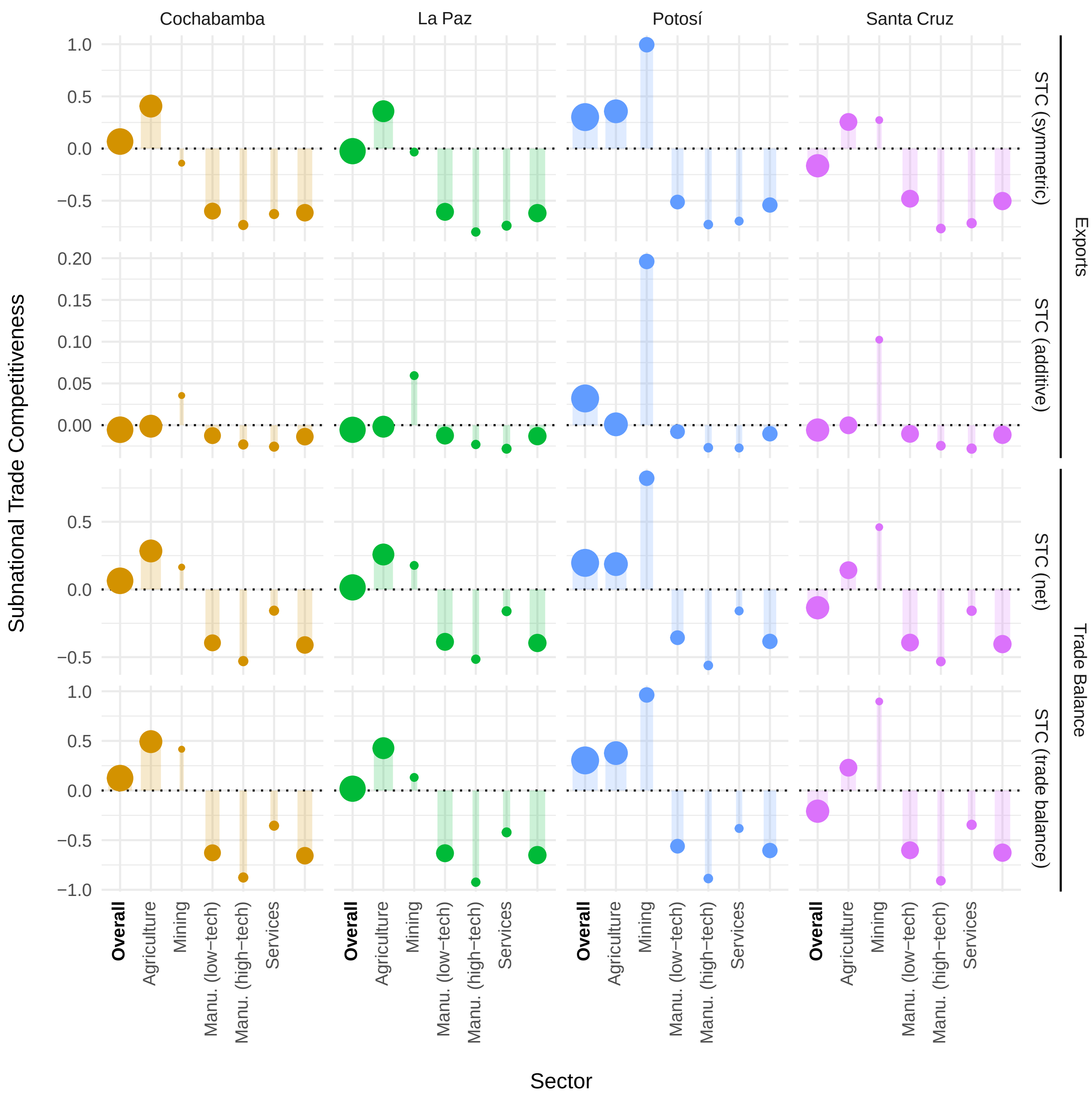

Note: Data for the year 2018.

G Used software and packages

We use the statistical software *R*[64]. We use version 4.2.1 at the time of submission. The following packages have been used for manipulating the data and the respective data analyses: *car* [version: 3.1-0][19] *countrycode* [1.4.0][3], *foreign* [0.8-82][63], *funtimes* [9.0][46], *ggh4x* [0.2.1][77], *ggtext* [0.1.1][81], *ggthemes* [4.2.4][4], *glue* [1.6.2][23], *haven* [2.5.0][78], *ipumsr* [0.5.0][20], *questionr* [0.7.7][5] *readxl* [1.4.0][79], *tidyverse* [1.3.2][80], *wbstats* [1.0.4][61], *xtable* [1.8-4][11], *zoo* [1.8-10][83].

References

1. ABS, Australian Bureau of Statistics. 2021. "Census 2006, 2011, and 2016.".
URL: *https://www.abs.gov.au/websitedbs/D3310114.nsf/home/About+TableBuilder*

2. ANSD, Agence nationale de la Statistique et de la Démographie (Senegal). 2013. "Grand Census 2013 of Population, Housing, Agruculture, Livestock, and Farming.".
URL: *https://international.ipums.org/*

3. Arel-Bundock, Vincent, Nils Enevoldsen, and CJ Yetman. 2018. "Countrycode: An R Package to Convert Country Names and Country Codes." *Journal of Open Source Software* 3 (28): 848.

4. Arnold, Jeffrey B. 2021. *Ggthemes: Extra Themes, Scales and Geoms for 'Ggplot2'.*

5. Barnier, Julien, François Briatte, and Joseph Larmarange. 2022. *Questionr: Functions to Make Surveys Processing Easier.*

6. BfS, Bundesamt für Statistik (Switzerland). 2020. "Die schweizerische Arbeitskräfteerhebung (SAKE) 2001-2018.".
URL: *https://www.bfs.admin.ch/bfs/de/home/dienstleistungen/forschung/zugang-anonymisierte-einzeldaten.html*

7. BPS, Badan Pusat Statistik (Indonesia). 2020. "National Labor Force Survey 2000-2015.".
URL: *https://www.bps.go.id/*

8. BUCREP, Bureau Central des Recensements et des Études de Population (Cameroon). 2005. "Third General Census of Population and Housing.".
URL: *https://international.ipums.org/*

9. CAPMAS, Central Agency for Public Mobilization and Statistics (Egypt). 2017. "Labor Force Survey (LFS) 2006-2017.".
URL: *http://erf.org.eg/data-portal/*

10. CSO, Czech Statistical Office. 2021. "Czech Labour Survey 2000-2019.".
URL: *https://www.czso.cz/csu/czso/4-contacts*

11. Dahl, David B., David Scott, Charles Roosen, Arni Magnusson, and Jonathan Swinton. 2019. *Xtable: Export Tables to LaTeX or HTML.*

12. DANE, Departamento Administrativo Nacional de Estadística de Colombia. 2021. "Gran Encuesta Integrada de Hogares (GEIH) 2007-2019.".
URL: *http://microdatos.dane.gov.co/index.php/catalog*

13. DESTATIS, Statitisches Bundesamt (Germany). 2020. "Mikrozensus 2000-2018.".
URL: *https://www.forschungsdatenzentrum.de/de/haushalte/mikrozensus*

14. DIGESTYC, Dirección General de Estadística y Censos de El Salvador. 2021. "Encuesta de Hogares de Propósitos Múltiples 2008-2016.".
URL: *http://digestyc.microdatahub.com/index.php/catalog*

15. DOS, The Hashemite Kingdom of Jordan, Department of Statistics (DOS). 2016. "Employment and Unemployment Survey (EUS) 2004-2016.".
URL: *http://erf.org.eg/data-portal/*

16. DOSM, Department of Statistics Malaysia. 2000. "2000 Population and Housing Census.".
URL: *https://international.ipums.org/*

17. Economic Research Forum. 2020. "ERF Micro Data Catalogue (NADA).".
URL: *http://erf.org.eg/data-portal/*

18. ELSTAT, Hellenic Statistical Authority. 2021. "Labour Force Survey 2006-2019.".
URL: *https://www.statistics.gr/en/public-use-files*

19. Fox, John, and Sanford Weisberg. 2019. *An R Companion to Applied Regression.* Third edition ed. Los Angeles: SAGE.

20. Greg Freedman Ellis, and Derek Burk. 2022. *Ipumsr: Read 'IPUMS' Extract Files.*

21. GSO, General Statistics Office of Vietnam. 2009. "Population and Housing Census 1999 & 2009.".
URL: *https://international.ipums.org/*

22. GSS, Ghana Statistical Service. 2021. "Ghana Living Standards Survey 2005, 2012, and 2017.".
URL: *https://www2.statsghana.gov.gh/nada/index.php/catalog*

23. Hester, Jim, and Jennifer Bryan. 2022. *Glue: Interpreted String Literals.*

24. IBGE, Instituto Brasileiro de Geografia e Estatística. 2021. "Pesquisa Nacional por Amostra de Domicílios (PNAD) 2002-2015.".
URL: *https://www.ibge.gov.br/estatisticas/sociais/educacao/9127-pesquisa-nacional-por-amostra-de-domicilios.html?=&t=microdados*

25. IHSI, Institute Haïtien de Statistique et d'Informatique. 2003. "Recensement General de la Population et de l'Habitat 2003.".
URL: *https://international.ipums.org/*

26. ILO, International Labour Organization. 2020. "Employment by Sex and Economic Activity – ILO Modelled Estimates, Nov. 2020 (Thousands) - Annual.".

27. INDEC, Instituto Nacional de Estadística y Censos de la República Argentina. 2021. "Encuesta Permanente de Hogares.".
URL: *https://www.indec.gob.ar/indec/web/Institucional-Indec-BasesDeDatos*

28. INE, Instituto Nacional de Estadística (Bolivia). 2021*a*. "Encuesta de Hogares 2004-2019.".
URL: *https://www.ine.gob.bo/index.php/censos-y-banco-de-datos/censos/bases-de-datos-encuestas-sociales/*

29. INE, Instituto Nacional de Estadística (Honduras). 2001*a*. "XVI Censo de Poblacion y V de Vivienda.".
URL: *https://international.ipums.org/*

30. INE, Instituto Nacional de Estadística (Spain). 2020. "Encuesta de Población Activa 2006-2019.".
URL: *https://www.ine.es/en/prodyser/microdatos_en.htm*

31. INE, Instituto Nacional de Estadística (Uruguay). 2021*b*. "Encuesta Continua de Hogares 2006-2019.".
URL: *http://www.ine.gub.uy/web/guest/encuesta-continua-de-hogares1*

32. INE, Instituto Nacional de Estadística (Venezuela). 2001*b*. "XIII Censo General de Población y Vivienda.".
URL: *https://international.ipums.org/*

33. INE, Instituto Nacional de Estatística (Portugal). 2011. "Receseamento Geral da População 2001 & 2011.".
URL: *https://international.ipums.org/*

34. INEC, Instituto Nacional de Estadística y Censos (Costa Rica). 2021*a*. "Encuesta Continua de Empleo 2010-2019.".
URL: *http://sistemas.inec.cr/pad5/index.php/catalog/REGECE*

35. INEC, Instituto Nacional de Estadística y Censos (Ecuador). 2021*b*. "Encuesta Nacional de Empleo, Desempleo y Subempleo 2007-2019.".
URL: *http://aplicaciones3.ecuadorencifras.gob.ec/BIINEC-war/index.xhtml*

36. INEC, Instituto Nacional de Estadística y Censos (Panama). 2021*c*. "Encuesta de Mercado Laboral 2012-2017.".
URL: *http://www.inec.gob.pa/dbnew/pass/indice.html*

37. INEGI, Geografía e Informática (Mexico), Instituto Nacional de Estadística. 2021. "Encuesta Nacional de Ocupación y Empleo (ENOE), 2005-2019.".
URL: *https://www.inegi.org.mx/programas/enoe/15ymas/?ps=Microdatos*

38. INEI, Instituto Nacional de Estadística e Informática de Perú. 2021. "Encuesta Nacional de Hogares 2004-2019.".
URL: *http://iinei.inei.gob.pe/microdatos/Consulta_por_Encuesta.asp*

39. INIDE, Instituto Nacional de Información de Desarrollo de Nicaragua. 2021. "Encuesta Nacional de Hogares sobre Medición de Nivel de Vida 2005, 2012, and 2014.".
URL: *http://www.inide.gob.ni/bibliovirtual/basesdatos.htm*

40. INS, Institut National de la Statistique (Guinea). 2014. "Third General Census of the Population and Inhabitants.".
URL: *https://international.ipums.org/*

41. INSAE, L'Institut National de la Statistique et de l'Analyse Economique (Benin). 2013. "Fourth Population and Habitation Census.".
URL: *https://international.ipums.org/*

42. INSEED, Institut National de la Statistique et des Etudes Economiques et Démographiques. 2010. "General Census of the Population and Habitat 2010.".
URL: *https://international.ipums.org/*

43. ISTAT, National Institute of Statistics (Italy). 2019. "Labour Force Survey 2014-2019.".
URL: *https://international.ipums.org/*

44. KLI, Korea Labor Institute. 2019. "Korean Labor & Income Panel Study 2000-2018.".
URL: *https://www.kli.re.kr/klips_eng/index.do*

45. LSB, Lao Statistics Bureau. 2005. "2005 Population and Housing Census.".
URL: *https://international.ipums.org/*

46. Lyubchich, Vyacheslav, Yulia R. Gel, and Srishti Vishwakarma. 2022. *Funtimes: Functions for Time Series Analysis*.

47. MDSF, Ministerio de Desarrollo Social y Familia de Chile. 2021. "Encuesta de Caracterizacion Socioeconomica Nacional (CASEN) 2000-2017.".
URL: *http://observatorio.ministeriodesarrollosocial.gob.cl/encuesta-casen*

48. Minnesota Population Center. 2019. "Integrated Public Use Microdata Series, International: Version 7.2.". Version Number: 7.2 type: dataset.
URL: *https://www.ipums.org/projects/ipums-international/d020.V7.2*

49. MoSPI, Ministry of Statistics & Programme Implementation (India). 2021. "Employment and Unemployment Survey, 2000, 2004, 2005, 2006, 2008, 2010, and 2012.".
URL: *http://microdata.gov.in/nada43/index.php/catalog/127/related_materials*

50. NBS, Tanzania National Bureau of Statistics. 2021. "Intergrated Labour Force Survey 2006 & 2014.".
URL: *https://nbs.go.tz/tnada/index.php/catalog/31*

51. NISR, National Institute of Statistics of Rwanda. 2012. "Population and Housing Census 2002 & 2012.".
URL: *https://international.ipums.org/*

52. NSA, Namibia Statistics Agency. 2021. "Labour Force Survey 2013 & 2016.".
URL: *https://nsa.org.na/microdata1/index.php/catalog*

53. NSC, National Statistical Committee of the Kyrgyz Republic. 2009. "Census of Population and Housing of the Kyrgyz Republic, 1999 & 2009.".
URL: *https://international.ipums.org/*

54. NSO, National Statistical Office of Mongolia. 2021. "Labour Force Survey 20003-2018.".
URL: *http://web.nso.mn/nada/index.php/catalog/LFS*

55. NSO, National Statistical Office of Thailand. 2000*a*. "The 2000 Population and Housing Census of Thailand.".
URL: *https://international.ipums.org/*

56. NSO, National Statistics Office of Papua New Guinea. 2000*b*. "National Census 2000.".
URL: *https://international.ipums.org/*

57. NSS, National Statistical Service of the Republic of Armenia. 2011. “The 2011 Population and Housing Census of the Republic of Armenia.”.
URL: *https://international.ipums.org/*

58. ONE, Oficina Nacional de Estadística de la República Dominicana. 2021. “Encuesta de Hogares de Propositos Multiples 2009, 2013, and 2015.”.
URL: *https://www.one.gob.do/*

59. ONS, Office for National Statistics (United Kingdom). 2021. “Quarterly Labour Force Survey 2000-2019.”.
URL: *https://beta.ukdataservice.ac.uk/*

60. PCBS, Palestinian Central Bureau of Statistics. 2008. “Labor Force Survey (LFS) 2008.”.
URL: *http://erf.org.eg/data-portal/*

61. Piburn, Jesse. 2020. *Wbstats: Programmatic Access to the World Bank API.* Oak Ridge, Tennessee: Oak Ridge National Laboratory.

62. PSA, Philippine Statistics Authority. 2010. “2010 Census of Population and Housing.”.
URL: *https://international.ipums.org/*

63. R Core Team. 2022*a*. *Foreign: Read Data Stored by ’Minitab’, ’S’, ’SAS’, ’SPSS’, ’Stata’, ’Systat’, ’Weka’, ’dBase’, ...*

64. R Core Team. 2022*b*. *R: A Language and Environment for Statistical Computing*. Vienna, Austria: R Foundation for Statistical Computing.

65. SCI, Statistical Center of Iran. 2011. “National Population and Housing Census 2006 & 2011.”.
URL: *https://international.ipums.org/*

66. Smits, Jeroen, and Iñaki Permanyer. 2019. “The Subnational Human Development Database.” *Scientific Data* 6 (1): 190038.

67. StatAustria, Statistik Austria. 2020. “Mikrozensus 2003-2019.”.
URL: *https://data.aussda.at/dataverse/statistikaustria*

68. StatBel, Belgian Statistical Office. 2020. “Labour Force Survey 2013-2019.”.
URL: *https://statbel.fgov.be/en/about-statbel/what-we-do/microdata-research*

69. StatEst, Statistikaamet (Estonia). 2020. “Labour Force Survey 2000-2019.”.
URL: *https://www.stat.ee/en/find-statistics/request-statistics/request-microdata-research*

70. STATIN, Statistical Institute of Jamaica. 2001. “Population Census 2001.”.
URL: *https://international.ipums.org/*

71. Stats SA, Statistics South Africa. 2021. “Quarterly Labour Force Survey 2000-2019.”.
URL: *https://www.datafirst.uct.ac.za/dataportal/index.php/catalog/499/get_microdata*

72. StatsBots, Statistics Botswana. 2011. “Population and Housing Census 2001 & 2011.”.
URL: *https://international.ipums.org/*

73. StatsCAN, Statistics Canada. 2021. “Labour Force Survey 2000-2019.”.
URL: *https://www.statcan.gc.ca/eng/help/microdata*

74. StatSVK, Statistical Office of the Slovak Republic. 2020. “Slovakian Labour Force Survey 2003-2019.”.
URL: *https://slovak.statistics.sk/wps/portal/ext/services/*

75. UNSD, United Nations Statistics Division. 2008. “International Standard Industrial Classification of All Economic Activities (ISIC), Rev.4.”.

76. USCB, U.S. Census Bureau. 2018. “American Community Survey 2000-2018.”.
URL: *https://international.ipums.org/*

77. van den Brand, Teun. 2021. *Ggh4x: Hacks for ’Ggplot2’.*

78. Wickham, Hadley, Evan Miller, and Danny Smith. 2022. *Haven: Import and Export 'SPSS', 'stata' and 'SAS' Files*.

79. Wickham, Hadley, and Jennifer Bryan. 2022. *Readxl: Read Excel Files*.

80. Wickham, Hadley, Mara Averick, Jennifer Bryan, Winston Chang, Lucy D'Agostino McGowan, Romain François, Garrett Grolemund, Alex Hayes, Lionel Henry, Jim Hester, Max Kuhn, Thomas Lin Pedersen, Evan Miller, Stephan Milton Bache, Kirill Müller, Jeroen Ooms, David Robinson, Dana Paige Seidel, Vitalie Spinu, Kohske Takahashi, Davis Vaughan, Claus Wilke, Kara Woo, and Hiroaki Yutani. 2019. "Welcome to the tidyverse." *Journal of Open Source Software* 4 (43): 1686.

81. Wilke, Claus O. 2020. *Ggtext: Improved Text Rendering Support for 'Ggplot2'*.

82. ZamStats, Zambia Statistics Agency. 2010. "2010 Census of Population and Housing.".
URL: *https://international.ipums.org/*

83. Zeileis, Achim, and Gabor Grothendieck. 2005. "Zoo: S3 Infrastructure for Regular and Irregular Time Series." *Journal of Statistical Software* 14 (6): 1–27.
